# Plecstatin inhibits hepatocellular carcinoma tumorigenesis and invasion through cytolinker plectin

**DOI:** 10.1002/1878-0261.70186

**Published:** 2025-12-30

**Authors:** Zuzana Outla, Jan Kosla, Magdalena Prechova, Lukas Frick, Patricia Bortel, Yasmin Borutzki, Andrea Bileck, Christopher Gerner, Samuel M. Meier‐Menches, Selma Osmanagic‐Myers, Martin Gregor

**Affiliations:** ^1^ Laboratory of Integrative Biology Institute of Molecular Genetics of the Czech Academy of Sciences Prague Czech Republic; ^2^ Institute of Neuropathology, University Hospital Zurich University of Zurich Switzerland; ^3^ Department of Analytical Chemistry University of Vienna Austria; ^4^ Institute of Inorganic Chemistry University of Vienna Austria; ^5^ Joint Metabolome Facility Medical University of Vienna and University of Vienna Austria; ^6^ Center for Pathobiochemistry and Genetics Medical University of Vienna Austria

**Keywords:** hepatocellular carcinoma, ODF2, organometallic compounds, plecstatin, plectin, therapeutic target

## Abstract

Plecstatin (PST) is a potent anticancer agent in preclinical models, yet its precise mechanism of action and molecular specificity regarding its main targets, plectin and outer dense fiber protein 2 (ODF2), remain incompletely understood. Here, we dissected PST's mode of action using knockouts of plectin (*PLEC*) and *ODF2* in SNU‐475 hepatocellular carcinoma (HCC) cells. PST suppressed anchorage‐independent growth and impaired 2D and 3D migration in a dose‐dependent manner in both wild‐type and *ODF2*‐deficient cells, but not in *PLEC*‐deficient cells, establishing plectin as the principal effector of PST's antitumor activity. Proteomic and functional analyses revealed that PST primarily disrupts cytoskeletal remodeling through plectin, while selectively affecting ciliogenesis‐related pathways linked to ODF2 loss. Deletion of either protein attenuated PST‐induced Ser51 phosphorylation of eIF2α, ATF4/GADD34 induction, and cytochrome c release, indicating cooperative involvement in integrated stress response (ISR). Correlative analysis of patient datasets confirmed associations between *PLEC* */ODF2* expression and ISR‐related gene signatures, supporting the clinical relevance of this pathway. Together, these findings identify plectin as a key target of PST in disrupting cytoskeletal integrity and establish plectin/ODF2 axis in PST‐driven stress adaptation in HCC.

## Graphical abstract

The ruthenium‐based metallodrug plecstatin exerts its anticancer effect in hepatocellular carcinoma (HCC) primarily through selective targeting of plectin. By disrupting plectin‐mediated cytoskeletal organization, plecstatin inhibits anchorage‐dependent growth, cell polarization, and tumor cell dissemination. The resulting activation of the integrated stress response (ISR) ultimately commits HCC cells to apoptosis.

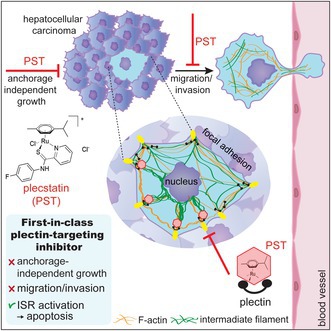

**Abbreviations**
ER: endoplasmic reticulum
HCC: hepatocellular carcinoma
ISR: integrated stress response
KO: knockout
MS: mass spectrometry
ODF2: outer dense fiber protein 2
PST: plecstatin
WT: wild‐type

## 1 Introduction

Hepatocellular carcinoma (HCC), the most common type of liver cancer, has a rising incidence worldwide and poses a major global health burden. To date, no effective targeted therapies exist for the key oncogenic drivers in HCC, such as *TERT*, *TP53*, fibroblast growth factor 19 (*FGF19*), *MYC*, or ß‐catenin (*CTNNB1*), all of which remain largely undruggable (reviewed in [1, 2]). For patients with advanced disease, systemic therapy remains the primary treatment strategy, including multikinase inhibitors, anti‐VEGF agents, and immune checkpoint inhibitors (reviewed in [3, 4]). However, these therapies are often associated with adverse side effects, limited tolerability, and only modest efficacy, highlighting the urgent need for more effective and better‐tolerated treatment options (reviewed in [3]).

An emerging class of anticancer strategies involves the targeting of cytoskeletal filaments and cytoskeleton‐associated proteins using metallodrugs, including platinum‐, gold‐, or ruthenium‐based compounds [5]. Among these, we have previously identified the novel *S,N*‐bidentate pyridinecarbothioamide ruthenium(arene) complex, termed plecstatin (PST), which has two main targets, plectin and the outer dense fiber protein 2 (ODF2), as identified by mass‐spectrometry (MS) target profiling [6]. PST is administered in a metal‐halogen prodrug form that, once activated by hydrolysis, preferentially in the cellular environment, binds its selective targets (Fig. S1A). We have recently demonstrated the potent antitumor efficacy of PST in various preclinical models of HCC [7]. PST inhibited the growth of subcutaneous Huh7 xenografts and *MYC*‐driven HCC tumors on a *TP53*‐deficient background. Importantly, PST also suppressed tumor dissemination, as evidenced by a significant reduction in lung invasion by HCC cells expressing RedFLuc‐GFP [7].

The primary PST target, plectin, is a cytoskeletal crosslinker (cytolinker) protein essential for cytoskeletal integrity, mediating the interconnection of intermediate filaments with actin, microtubules, and cell adhesion complexes [8, 9, 10]. Through its role in cytoskeletal organization, plectin regulates cancer cell migration, invasion [11, 12, 13, 14, 15], and xenograft tumor growth [11, 16, 17], positioning it as a promising molecular target in the context of tumor cell mechanics [17]. Plectin is frequently upregulated at the mRNA and protein levels in multiple tumor types, including HCC, where it is often amplified or overexpressed (reviewed in [8, 18, 19]).

At the molecular level, we previously showed that PST disrupts plectin‐mediated cytoskeletal crosslinking and force transmission to adhesion sites, thereby interfering with mechanosignaling pathways that promote tumor growth and migration [6, 7, 9]. Apart from monoclonal antibodies targeting surface plectin (reviewed in [17]), PST remains hitherto the only compound known to modulate plectin functions, effectively mimicking genetic plectin depletion [8, 9]. The translational relevance of these findings is underscored by HCC patient data, where elevated plectin expression correlates with more aggressive disease and poor prognosis [7].

Given these highly beneficial effects of PST in suppressing liver tumorigenicity, it is imperative to elucidate its detailed mode of action and specificity in the context of liver carcinoma to facilitate the clinical translation of this metal‐based therapeutic. To identify its selective targets, we employed an integrated proteomic‐based target‐response profiling strategy, which involved exposing PST to whole nondenaturing cancer cell lysates followed by affinity enrichment using a PST‐immobilized solid support. This approach allowed us to assess target selectivity under near‐physiological conditions (see Fig. S1B adopted from [6]). Using this method, plectin emerged as the top binding target of PST, with only one other protein, ODF2, displaying a comparable binding probability [6]. The *ODF2* gene encodes multiple isoforms, including isoform 9, which corresponds to the centrosomal protein cenexin1, a well‐established regulator of primary cilia formation [20, 21].

Building on these findings, we aimed to dissect the relative contributions of plectin and ODF2 to the anticancer effects of PST through functional target validation. In doing so, we also uncover a previously unrecognized role of ODF2 in HCC‐related processes, including tumorigenesis, collective invasion, and stress signaling. To this end, we present the first systematic analysis of PST responses in gene‐edited human HCC cells lacking either plectin or ODF2. This approach enabled us to determine whether the presence of these two identified interactors is required for PST to exert its antitumor activity.

## 2 Materials and methods

### 2.1 Patient analysis data mining

To investigate the association of *ODF2* mRNA expression with gene signatures and clinical parameters in HCC patients, mRNA expression datasets were obtained from online repositories including Gene Expression Omnibus (GEO), ArrayExpress, and the Cancer Genome Atlas. For Affymetrix whole‐genome microarray data, raw CEL files were processed using the SCAN (single channel array normalization) method [22], and samples with median GNUSE quality scores [23] above 1.25, as computed by the ‘frma’ Bioconductor package, were excluded. For all other datasets, processed data from the original authors were used. Cases of fibrolamellar carcinoma were removed [24]. Across all platforms, probe or gene IDs were mapped to Entrez gene identifiers. For microarray platforms containing multiple alternative probes or probe sets per gene, the probe showing the greatest variance in expression among HCC samples was selected. Meta‐analyses across datasets were computed with the ‘metafor’ R package [25] using a random‐effects model and the DerSimonian‐Laird estimator. The tumor, node, metastasis (TNM) analysis comprised datasets GSE14520, iCOD [26], GSE76427, GSE44074, GSE36376, and TCGA‐LIHC from Affymetrix HuGeneFL, Affymetrix HT_HG‐U133A, and Affymetrix HG‐U133 Plus 2.0, Illumina HumanHT‐12 V4.0, and Illumina HiSeq platforms. Collections of gene signatures were obtained from the Molecular Signatures Database (MSigDB [27, 28]), and used to produce quantitative gene set enrichment scores with the ‘GSVA’ (Gene Set Variation Analysis) Bioconductor package [29]. To assess the correlation of ODF2 expression with selected genes and pathways in human HCC, 15 datasets with extensive whole‐genome RNA expression data were chosen (TCGA LIHC, E_TABM_36, GSE14520, GSE15765, GSE16757, GSE25097, GSE65485, GSE50579, GSE45436, GSE62232, GSE9843 [30], iCOD [31], GSE63898, GSE64041 [32], GSE76297), and gene expression values were batch‐adjusted using the ComBat method [33] as implemented by the ‘sva’ r package. The Kaplan–Meier survival analysis of ODF2 expression was based on three datasets for which overall survival data were available: E‐TABM‐36, GSE14520, GSE364, GSE45114, GSE62232, GSE76427, ICGC‐LIRI‐JP, and TCGA‐LIHC.

### 2.2 Cell culture

The human HCC cell line SNU‐475 (RRID:CVCL_0497) and 16 independent PCR systems were investigated using the AmpFlSTR^®^ Identifiler^®^ Plus PCR Amplification Kit (Thermo Fisher Scientific, Prague, Czech Republic), as described in SOP_APG_Zelllinienauthentizität_3.0_15.04.2024. The results of the cell line analysis were then compared with those in the online databases of the DSMZ (https://www.dsmz.de/services/human‐and‐animal‐cell‐lines) and the Cellosaurus database (https://web.expasy.org/cellosaurus). The SNU‐475 cell line was confirmed to be free of mycoplasma contamination using regular PCR testing. The cell line was maintained in DMEM supplemented with 10% fetal bovine serum (FBS, Gibco, Waltham, MA, USA, Thermo Fisher Scientific) and 1% penicillin‐streptomycin (Sigma‐Aldrich, St. Louis, MO, USA) in a 5% CO_2_/air humidified atmosphere at 37 °C.

### 2.3 Plecstatin treatment

Plecstatin (PST) was synthesized at the Faculty of Chemistry, University of Vienna, as previously described [6]. Stocks were dissolved in dimethylsulfoxide to a concentration of 4 mm and diluted in complete medium or DMEM only.

### 2.4 Preparation of protein lysates and immunoblot analysis

7 × 10^5^ cells were seeded on a P100 plate coated with fibronectin (Sigma‐Aldrich, St. Louis, MO, USA) and incubated for 24 h. For PST treatment, PST in a final concentration of 15 or 33 μm was added 8 h after cell seeding. Cells were rinsed with PBS and lysed in RIPA buffer (20 mm Tris pH 7.4, 150 mm NaCl, 0.1% sodium dodecyl sulfate, 0.5% Na‐Deoxycholate, 1% Triton X‐100), supplemented with protease and phosphatase inhibitors (Roche, Basel, Switzerland). Protein concentrations were determined using a BCA Protein Assay Kit using the manufacturer's protocol (Thermo‐Fisher Scientific). Proteins were separated by SDS/PAGE and transferred to nitrocellulose membranes. Membranes were incubated with 5% BSA in PBST to block nonspecific antigen interactions and subsequently incubated with primary and secondary antibodies (Table S1). Signals were detected and quantified using an Odyssey imaging system and image studio 6.0 software, LI‐COR Biosciences, Lincoln, NE, USA.

### 2.5 Anchorage‐independent growth in soft agar

Soft agar assay was performed as previously described [7]. Briefly, the wells of a 6‐well plate were coated with 1.5 mL of 0.5% low‐gelling temperature agarose (Sigma‐Aldrich) solution dissolved in serum‐free DMEM. After solidification in RT, 2 × 10^3^ SNU‐475 cells were suspended in 1 mL of 0.35% agarose in complete DMEM, seeded on top of 0.5% agarose, and overlaid with 200 μL of complete DMEM. Cells were cultured for 28 days at 37 °C and 5% CO_2_, and the culture medium was exchanged once a week. For PST treatment, PST was added to the 0.35% agarose mixture and the overlaying medium in the indicated concentration and was replenished once a week. The colonies were stained and fixed with 0.01% crystal violet in 4% PFA in PBS and captured by Carl Zeiss Axio.Zoom V.16 microscope with PlanNeoFluar Z 1.0×/0.25 NA objective. The size and area of colonies were analyzed from binary masks created using a custom‐made imagej macro.

### 2.6 Cell morphology during random migration

Random migration assay was performed as previously described [7]. Briefly, SNU‐475 cells were seeded at a total number of 1 × 10^3^ onto a well of a 6‐well plate containing fibronectin‐coated coverslips. Cells were fixed 20 h after seeding with prewarmed 4% PFA in PBS for 10 min at RT and permeabilized with 0.2% Triton in PBS for 5 min. For PST treatment, PST was added 4 h before fixation in the indicated concentration. The fixed cells were stained with phalloidin and DAPI for 1 h at RT and imaged using a Leica DM6000 microscope and classified as polarized, intermediate, or round.

### 2.7 Scratch wound‐healing assay

Scratch wound‐healing assay was performed as described in [7]. Briefly, 1 × 10^5^ SNU‐475 cells were plated onto a well of 24‐well plates in triplicate and incubated under standard conditions overnight to achieve 100% confluence. Before imaging, PST was added for 6 h in the indicated concentration. Using a 200‐μL pipette tip, a linear scratch was made, running the length of each well. Subsequently, cells were washed with PBS and complete DMEM. To assess collective cell migration kinetics, 18‐h movies with a 20‐min time‐lapse interval were recorded using brightfield video microscopy obtained with a Leica DMi8 with HCX PL FLUOTAR L 20×/0.40 NA objective (37 °C and 5% CO_2_ in a humidified atmosphere; OKOlab, Naples, Italy). Individual fields of view were monitored in each well. The migration speed was calculated from the change of wound area between the initial and final time point, measured using imagej.

### 2.8 Spheroid invasion assay

Spheroid invasion assay was performed as described in [7]. Briefly, 5 × 10^5^ SNU‐475 cells were grown for 2 days in Microtissues^®^ 3D Petri Dish^®^ (Sigma Aldrich) according to the manufacturer's protocol. Formed spheroids were then embedded in 1.5 mg·mL^−1^ Collagen I (Thermo‐Fisher Scientific) solution containing DMEM, 5% NaHCO_3_, and 10% FBS. For PST treatment, PST was added to the collagen mixture and into the overlaying DMEM in the indicated concentration. Using a 96‐well plate, one spheroid was embedded per well in 40 μL of collagen mixture, overlayed with 40 μL of collagen mixture, and once collagen polymerized, 80 μL of DMEM was added to the wells. Images were taken after 3 days using a Leica DMi8 microscope with HC PL FLUOTAR 5×/0.25 NA objective. The invaded area was calculated as the percentage of the initial spheroid area using imagej.

### 2.9 Matrigel transwell invasion assay

Matrigel transwell invasion assay was performed as described in [7]. Briefly, 3 × 10^4^ SNU‐475 cells were seeded in serum‐free DMEM on the Matrigel‐coated transwell inserts at 37 °C for a minimum of 2 h and allowed to migrate for 20 h toward the lower chamber containing 10% FBS as an attractant. Inserts with migrated cells were washed three times with PBS and fixed with 4% PFA. After staining with 0.02% crystal violet solution in 10% ethanol for 40 min at RT, cells on the bottom surface of the inserts were imaged using a Leica DMi8 microscope with N PLAN 10×/0.25 NA objective.

### 2.10 Proteomic analysis of SNU‐475 cell culture

The cells were processed according to a nucleocytoplasmic fractionation protocol as previously described [6]. LC–MS/MS analyses and protein identification were performed as described in [7, 34]. All steps of the sample collection process were performed on ice.

#### 2.10.1 Sample preparation

1.5 × 10^6^ SNU‐475 cells were seeded in P100 plates. The cells were processed according to a nucleocytoplasmic fractionation protocol as previously described [6, 7]. The medium was removed, and the cells were washed twice with 1× PBS (3 mL). Then, PBS was thoroughly removed. Isotonic lysis buffer (10 mm HEPES, 10 mm NaCl, 3.5 mm MgCl_2_, 1 mm EGTA, 0.25 m Sucrose, 0.5% Triton X‐100) containing protease inhibitors (1% PMSF, Sigma, and 1% protease and phosphatase inhibitor cocktail, Roche) was added to the wells; the cells were scraped off and transferred into labeled 15 mL Falcon tubes. The cellular membrane was ruptured using shear stress by pressing the cell suspension through a syringe 9–12 times. Membrane rupture with intact nuclei was monitored under a microscope. After centrifugation (960 ***g***, 5 min), the supernatant containing the cytoplasmic proteins (soluble fraction referred to in the text as cytosolic) was transferred into ice‐cold ethanol (1 : 5, HPLC grade) and precipitated overnight at −20 °C. The pellet containing the nuclei was incubated with a hypertonic solution (10 mm Tris–HCl, 1 mm EDTA, and 0.5 m NaCl) and subsequently with 10 mm Tris–HCl, 1 mm EDTA, and 0.5% Triton X‐100 buffer containing protease inhibitors (1% PMSF, Sigma, and 1% protease and phosphatase inhibitor cocktail, Roche). Solubilization was assisted by an ultrasonic rod. After centrifugation (960 ***g***, 5 min), the solubilized proteins containing mainly cytoskeletal, nuclear, and mitochondrial‐residual proteins (insoluble fraction referred to in the text as cytoskeleton‐enriched fraction) were also transferred into ice‐cold ethanol (1 : 5, HPLC grade) and precipitated overnight at −20 °C. The precipitated proteins were finally pelleted by centrifugation (5000 ***g***, 30 min, 4 °C); the solution was decanted, and the pellet was dried under vacuum. The protein fractions were pelleted, dried, and solubilized in sample buffer (7.5 m urea, 1.5 m thiourea, 4% CHAPS, 0.05% SDS, and 100 mm dithiothreitol) before the total protein amount was determined by means of a colorimetric Bradford assay. A total of 20 μg protein per sample was then tryptically digested. This involved reduction with dithiothreitol, alkylation with iodoacetamide, and digestion with trypsin/Lys‐C according to a filter‐assisted protein digestion (FASP) protocol [6]. The obtained peptide samples were dried in a SpeedVac.

#### 2.10.2 LC–MS/MS analyses

LC–MS/MS analyses were performed employing a timsTOF Pro mass spectrometer (Bruker Daltonics, Bremen, Germany) hyphenated with a Dionex UltiMateTM 3000 RSLCnano system (Thermo Scientific, Bremen, Germany). Samples were analyzed in data‐dependent acquisition mode by label‐free quantification (LFQ) shotgun proteomics. The injection volume was 2 μL. Samples were loaded on an AcclaimTMPepMapTM C18 HPLC precolumn (2 cm × 100 μm, 100 Å, Thermo Fisher Scientific™, Vienna, Austria) at a flow rate of 10 μL·min^−1^ MS loading buffer. After trapping, peptides were eluted at a flow rate of 300 nL·min^−1^ and separated on an Aurora series CSI UHPLC emitter column (25 cm × 75 μm, 1.6 μm C18, Ionopticks, Fitzroy, Australia) applying a gradient of 8–40% mobile phase B (79.9% ACN, 20% H_2_O, 0.1% FA) in mobile phase A (99.9% H_2_O, 0.1% FA) over 95 min.

#### 2.10.3 Protein identification

Protein identification was performed via maxquant (version 1.6.17.0) employing the Andromeda search engine against the UniProt Database (version 11/2021, 20 375 entries). A mass tolerance of 20 ppm for MS spectra and 40 ppm for MS/MS spectra, a PSM‐, protein‐, and site‐false discovery rate (FDR) of 0.01, and a maximum of two missed cleavages per peptide were allowed. Match‐between‐runs was enabled with a matching time window of 0.7 min and an alignment time window of 20 min. Oxidation of methionine and N‐terminal protein acetylation were set as variable modifications. Carbamidomethylation of cysteine was set as a fixed modification. Global proteome data analysis was performed via perseus (version 1.6.14.0). Proteins with at least 60% quantification rate in at least one group were considered for analysis. A two‐sided Student's *t*‐test with *S*_0_ = 0.1 and an FDR cutoff of 0.05 was applied for identifying multiple testing‐corrected significantly regulated proteins.

### 2.11 Antibodies and reagents

For further details on primary and secondary antibodies used in this study, see Table S1. The following reagents were used: AF‐Phalloidin 594 (Invitrogen Life Technologies, Carlsbad, CA, USA), mitotracker red (Thermo‐Fisher Scientific), low‐gelling‐temperature agarose (Sigma‐Aldrich), Collagen I Rat Protein (Thermo‐Fisher Scientific), Crystal Violet solution (Sigma‐Aldrich), DAPI (AppliChem, Darmstadt, Germany), Fibronectin (FN) from bovine plasma (Sigma), Matrigel Basement Membrane Matrix (Corning, NY, USA).

### 2.12 CRISPR‐mediated targeting of plectin and ODF2

*PLEC* KO cell lines were generated using the CRISPR/Cas9 system, by targeting exon 6 of *PLEC* (Ensembl Plec‐205), which encodes the essential ABD and is shared among plectin isoforms, as described previously [7, 9]. *ODF2* KO SNU‐475 cell lines were generated using the CRISPR/Cas9 system by targeting exon 6 of *ODF2* (Ensembl ODF2‐201), which is shared among ODF2 isoforms. A single‐guided RNA (sgRNA) targeting *ODF2* (CAACATCGAGCGCATGAAGG) was designed using the web tool crispor [35]. DNA sequences coding guide RNA targeting sequences and crRNA array were synthesized and subcloned into a modified version of a px330‐Cas9‐Venus [36] using the BbsI restriction site, where Cas9 was fused to monomeric Venus as a selection marker. For WT isogenic control cell generation, the px330‐Cas9‐Venus vector lacking the guide RNA targeting sequence was used. The vectors were transiently transfected into cells using Lipofectamine^®^ LTX with Plus™ Reagent (Invitrogen Life Technologies, Carlsbad, CA, USA) according to the manufacturer's instructions. Cells were incubated with the transfection complexes for 48 h, followed by fluorescence‐activated cell sorting (FACS). Single‐cell clones were FACS sorted into 96‐well plates, and *ODF2* knockout was confirmed by DNA sequencing, immunofluorescence staining, and immunoblot analysis.

### 2.13 Immunofluorescence and analysis of ciliogenesis

Immunofluorescence was performed according to our established procedures as described in [7]. For assessment of ciliogenesis 1 × 10^5^ SNU‐475 cells were seeded onto a well of 24‐well plate containing fibronectin‐coated coverslips and incubated under standard conditions overnight to achieve 100% confluence. Next, cells were starved with serum‐free DMEM for 48 h. For PST treatment, PST was added together with the serum‐free DMEM in the indicated concentration. Cells were fixed with 4% PFA for 10 min at RT and permeabilized with 0.2% Triton X‐100 in PBS for 5 min at RT. To detect cilia, cells were immunolabeled using ARL13B antibody and nuclei were counterstained with DAPI. Cells were imaged using a Leica sp8 confocal microscope with HC PL APO 63×/1.40 NA immersion oil objective. The percentage of ciliated cells was calculated manually using lasx software.

### 2.14 Immunofluorescence analysis of cytochrome c release

5 × 10^4^ SNU‐475 cells were seeded per well of a 24‐well plate containing fibronectin‐coated coverslips and incubated for 24 h under standard conditions. For PST treatment, PST in a final concentration of 15 μm was added 8 h after cell seeding. 24 h after cell seeding, cells were incubated with Mitotracker Red FM Dye (Thermofisher) for 30 min at 37 °C. Cells were then fixed with 4% PFA for 10 min at RT followed by permeabilization with 0.2% Triton X‐100 in PBS for 5 min at RT. Cells were immunolabeled using a cytochrome c antibody, and nuclei were counterstained with DAPI. Cells were imaged using a Leica sp8 confocal microscope with HC PL APO 63×/1.40 NA immersion oil objective. The percentage of cytochrome‐releasing cells showing no overlap of mitotracker and cytochrome c signals was calculated manually using lasx software.

### 2.15 Statistical analyses

Graphs and statistical tests were performed using graphpad prism (GraphPad Software, Inc., La Jolla, CA, USA) except for patient analyses and proteomics of SNU‐475 cell cultures (see details in the corresponding section). The effect of genotype and treatment in *PLEC* KO and *ODF2* KO cells was tested using two‐way ANOVA, where comparison of individual experimental groups with the control group was performed using Sidak's multiple comparison test. Data distribution was assumed to be normal, but this was not formally tested. Statistical significance was determined at the level of *P < 0.05, **P < 0.01, ***P < 0.001. The number of independent experiments (*N*), number of data points (*n*), and the statistical tests used are specified for individual experiments in the figure legends.

## 3 Results

### 3.1 ODF2 emerges as a potential mediator of PST anticancer activity in HCC

**Fig. 1:**
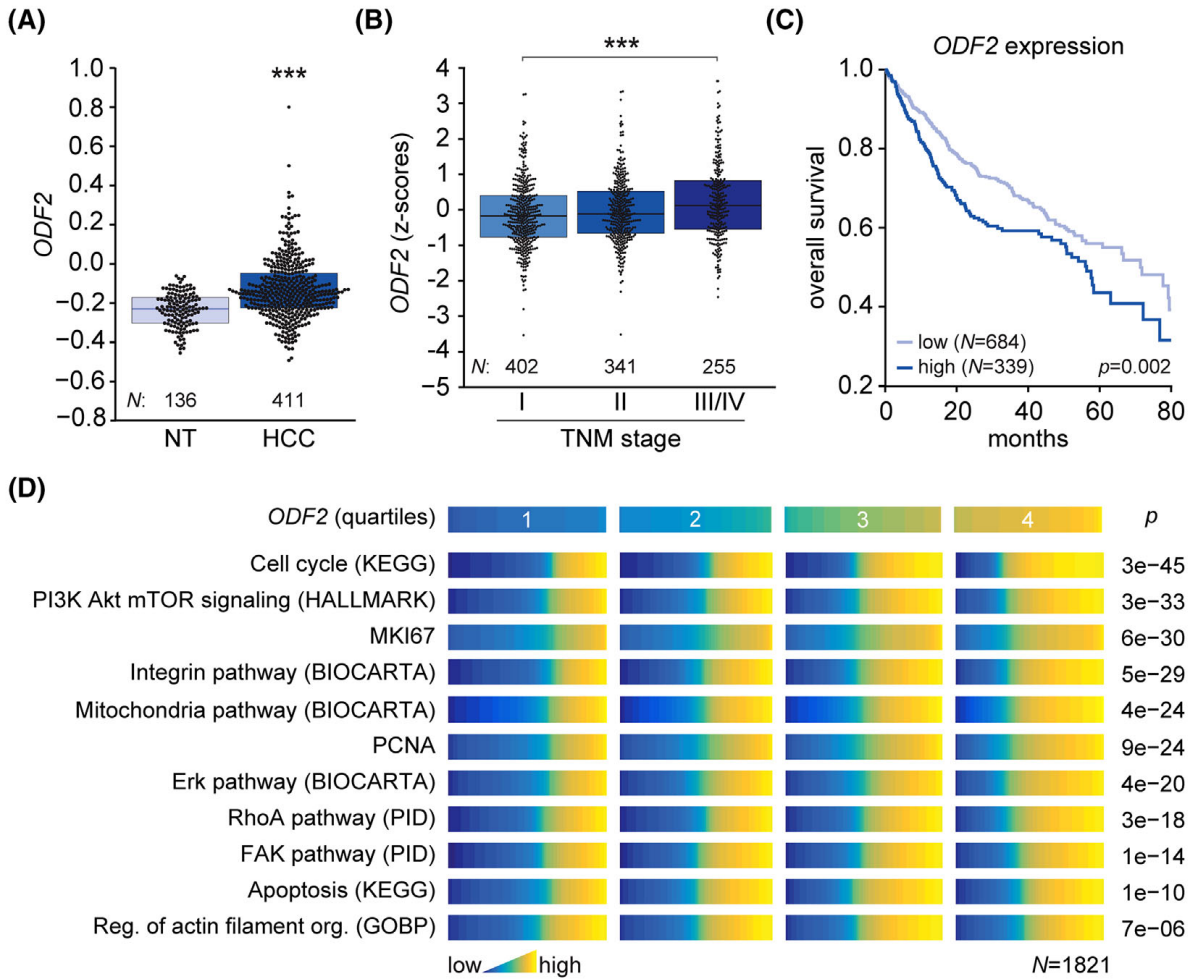
Increased ODF2 levels are associated with hepatocellular carcinoma (HCC) progression and poor prognosis. (A) Analysis of *ODF2* mRNA expression in non‐tumor (NT) and tumor tissue of liver hepatocellular carcinoma (HCC). The meta‐cohort includes 12 different datasets (for details, see Section 2). Scattered boxplots show individual data points, median, 25th, and 75th percentile; *N* = 547. Mann–Whitney test; ***P < 0.001. (B) Relative *ODF2* mRNA expression in samples collected from HCC patient meta‐cohort clustered based on tumor, node, metastasis (TNM) classification (stage I–IV). The meta‐cohort includes six different datasets from five platforms. The numbers of participants per stage are indicated in the graph. Scattered boxplots show individual data points, median, 25th, and 75th percentile; *N* = 998. Mann–Whitney test; ***P < 0.001. (C) Kaplan–Meier curve of overall survival of HCC patients with low *ODF2* (lower two tertiles, *N* = 684) and high *ODF2* (top tertile, *N* = 339) mRNA expression. *N* = 1023. Log‐rank test; *P* = 0.002. (D) Association of selected signatures compiled with *ODF2* mRNA expression in HCC patients. The color panel shows the levels of selected signatures in patients grouped into quartiles of *ODF2* gene expression levels. *N* = 1821. The analysis is based on data from gene set variation analysis, used to produce quantitative enrichment scores for all gene sets from msigdb in pooled and batch‐adjusted data. *P*‐values represent the result of a *chi*‐square test (for categorical data) or analysis of variance (for numerical data such as gene signature expression levels). *ODF2*, outer dense fiber protein 2; PST, plecstatin.

To investigate the molecular mechanism of PST anticancer effects, we first examined whether, similar to its known target plectin [7], the expression of *ODF2* is altered in HCC. To this end, we analyzed the *ODF2* gene expression levels across a publicly available meta‐cohort comprising 12 independent datasets of HCC patients. *ODF2* expression was elevated in tumor tissues and correlated with advanced tumor, node, metastasis (TNM) stage and poor overall survival (Fig. 1A–C, Fig. S1C), suggesting a role in tumor progression and metastasis. Consistent with this notion, PST has been shown to suppress tumor dissemination both *in vitro* and *in vivo*, as demonstrated by lung colonization assays using Huh7 and SNU‐475 hepatocellular carcinoma cells [7], which pose different mutations and represent different HCC molecular subtypes (Table S2).

We next explored the relationship between *ODF2* expression and HCC‐relevant transcriptional programs. Patients were stratified into quartiles based on *ODF2* expression levels, and enrichment of selected gene signatures associated with HCC carcinogenesis was assessed. High *ODF2* expression levels were significantly associated with proliferation‐related signatures (including cell cycle, PI3K Akt mTOR, and mKI67), mitochondrial and apoptotic signaling, as well as key mechanosensing‐related pathways (integrin, RhoA, Erk, FAK, and regulation of actin filament organization) (Fig. 1D). Together, these findings point to a previously underappreciated role for ODF2 in cancer progression, and suggest that it may act as an additional effector or modulator of PST's antitumor activity; warranting further investigation into whether PST's effects are fully plectin‐dependent.

Finally, we assessed the expression patterns of both plectin and ODF2 across the HCC tumor microenvironment using the Liver Single Cell Atlas from the University of Hong Kong (https://patholiver.hku.hk/liverp/), which includes data from 24 patients [37]. This analysis revealed that plectin (28.3%) and ODF2 (13.67%) are predominantly expressed in malignant cell populations (Fig. S2). ODF2, which exhibited overall lower expression levels, was also notably abundant in dendritic cells (DC, 14.57%; Fig. S2). Together, these data suggest that the principal targets of PST within the HCC tumor microenvironment are likely malignant cells, although contributions from stromal and immune compartments cannot be excluded.

### 3.2 Selective loss‐of‐function reveals plectin as the critical mediator of PST's antitumorigenic effects

To assess the individual contributions of plectin and ODF2 to PST‐mediated antitumorigenic activity, we selected HCC cell line SNU‐475, which harbors mutations in the *TP53* and *TERT* genes (Cellosaurus [38]). These represent two of the most frequent genomic alterations in HCC, occurring in 18.7–48% and 14.9–60% of patients, respectively [39, 40]. Furthermore, Nearest Template Prediction based on DepMap RNA expression profiles classified SNU‐475 cells as belonging to the highly invasive G3 subtype of Dr Boyault's HCC classification (Table S2, and see [41]). Both plectin and ODF2 exhibited significantly elevated expression in this G3 subtype, as well as in Dr Chiang's ‘proliferation’ subclass [30], further supporting the use of this cell line for dissecting PST‐mediated effects (Fig. S3A). Moreover, ODF2 expression levels showed a significant correlation with *TP53* mutation status in HCC cell lines, consistent with *TP53* being the major driver mutation in SNU‐475 (Fig. S3B).

We employed a loss‐of‐function dependency approach based on the rationale that reduced sensitivity to PST upon gene knockout (KO) would indicate that the respective protein is required for PST's activity. Using CRISPR/Cas9‐mediated gene editing, we generated *ODF2* KO cells and corresponding isogenic controls (referred to as wild‐type, WT), and included previously established *PLEC* KO cells [7] in parallel (Fig. S4A). Successful gene ablation was verified by immunoblotting and immunofluorescence analyses, both confirming the complete loss of plectin or ODF2 expression in the respective KO cell lines (Fig. S4B,C).

**Fig. 2:**
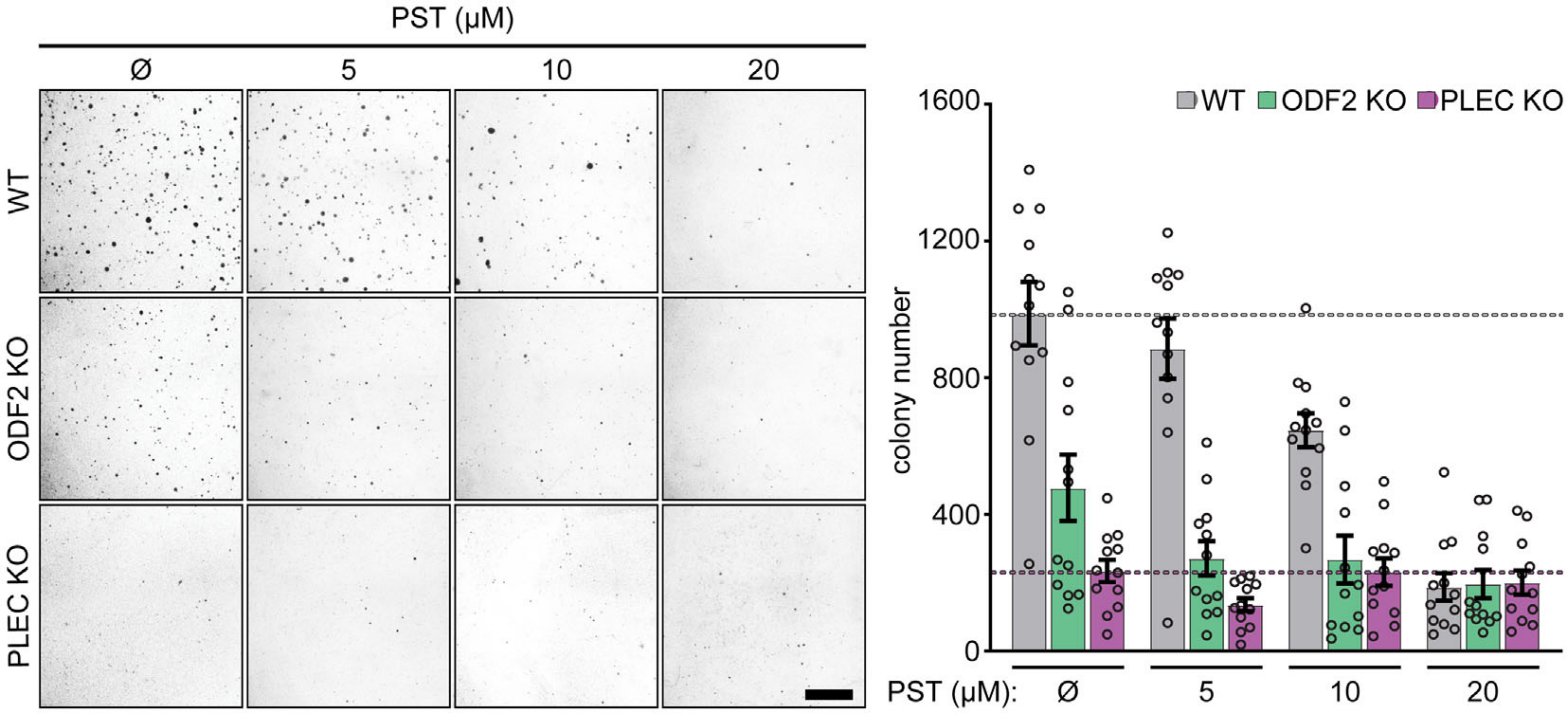
Plectin‐mediated effect of PST treatment on tumorigenic potential of HCC cells. Representative images of colonies from WT, *ODF2* KO, and *PLEC* KO SNU‐475 cells grown in soft agar, treated with the indicated concentrations of PST. Scale bar 2 mm. The graph shows the quantification of the cell colony numbers. Dotted lines depict mean control WT (gray) and *PLEC* KO (magenta) colony number. Data are shown as mean ± SEM; dots, agar wells; *N* = 4. Two‐way ANOVA: treatment (PST concentration, *P* < 0.0001), genotype (WT, *ODF2* KO, *PLEC* KO, *P* < 0.0001), and a treatment × genotype interaction (*P* < 0.0001). HCC, hepatocellular carcinoma; KO, knockout; *ODF2*, outer dense fiber protein 2; *PLEC*, plectin; PST, plecstatin; WT, wild‐type.

For all experiments, we used PST‐1 (hereafter referred to as PST) within a concentration range of 5–40 μm, previously established to maintain biological activity while exerting minimal cytotoxicity [7]. We first evaluated PST responses in the generated KO cell lines using a soft agar assay, a standard approach to assess anchorage‐independent tumorigenic growth of cancer cells. While *ODF2* KO cells exhibited reduced basal colony formation, their dose‐dependent response to PST closely mirrored that of WT cells, suggesting that ODF2 is dispensable for PST‐mediated growth inhibition (Fig. 2). In contrast, *PLEC* KO SNU‐475 cells were virtually unresponsive to PST. They formed only minimal colonies regardless of treatment dose, resembling WT cells treated with the highest (20 μm) PST dose (Fig. 2).

Consistent with our previous observations, PST did not affect colony size in any of the tested cell lines, suggesting that its inhibitory effects are restricted to early colony initiation and/or survival, rather than impacting subsequent colony outgrowth (Fig. S4D). Interestingly, both *PLEC* and *ODF2* KO cells showed reduced anchorage‐independent growth compared to WT controls under untreated conditions, indicating that both proteins contribute to intrinsic tumorigenic potential independently of PST. Together, these findings demonstrate that the antitumorigenic efficacy of PST is primarily mediated through plectin inhibition, while ODF2 does not appear to play a critical role in this context.

### 3.3 Plectin, not ODF2, mediates PST‐driven inhibition of HCC migration and invasion

**Fig. 3:**
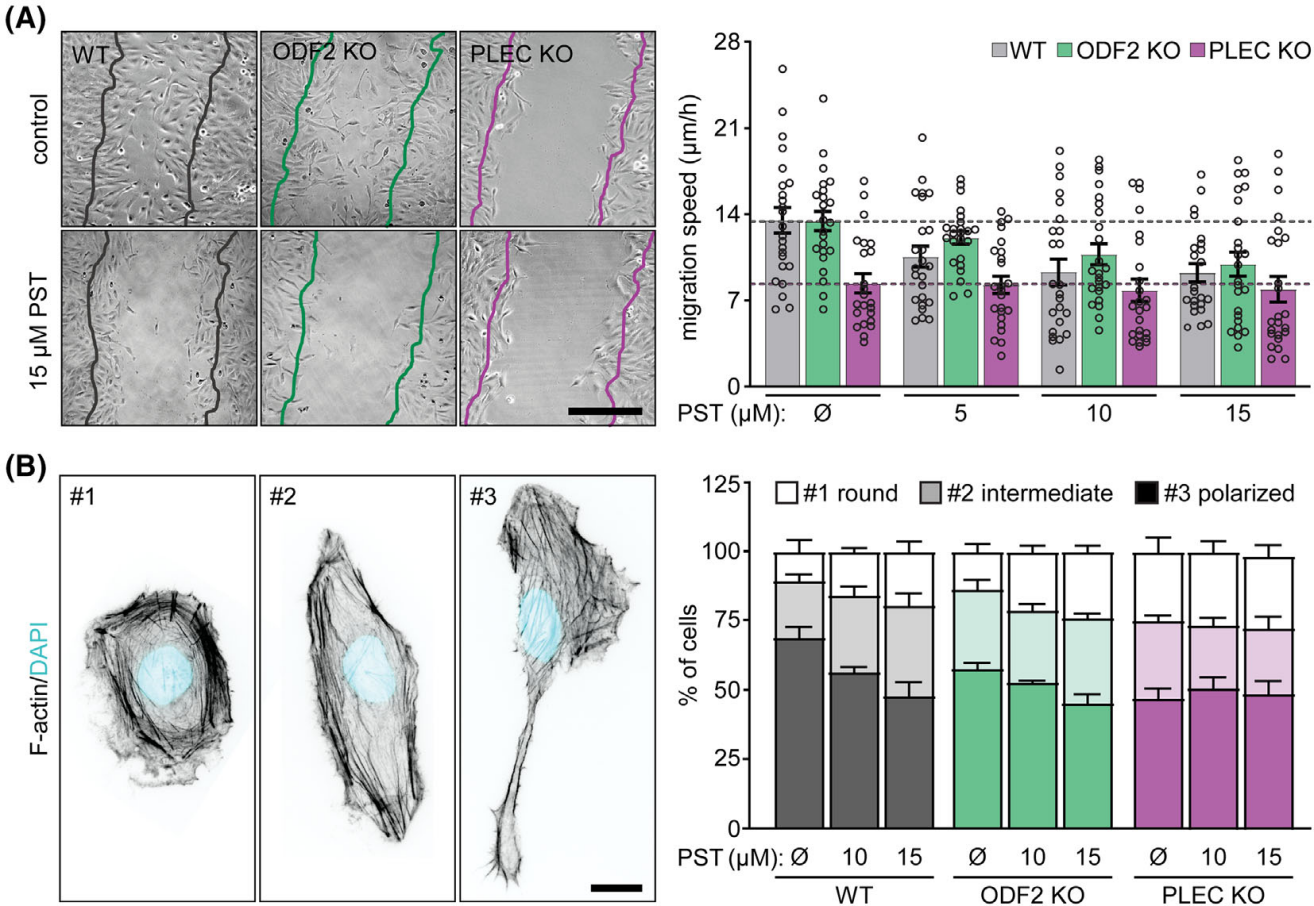
Plectin‐mediated effect of PST treatment on HCC cell polarity and migration potential. (A) Representative phase contrast images of WT, *ODF2* KO, and *PLEC* KO SNU‐475 cells migrating in the scratch‐wound assay for 18 h, pre‐treated with indicated concentrations of PST for 6 h. Lines, wound edge from the initial time point. Scale bar, 250 μm. Graph, migration speed. Dotted lines, mean control WT (gray) and *PLEC* KO (magenta) migration speeds. Data are shown as mean ± SEM; dots, fields of view; *N* = 5. Two‐way ANOVA: Treatment (PST concentration, *P* < 0.001) and genotype (WT, *ODF2* KO, *PLEC* KO, *P* < 0.0001). (B) Representative confocal images of F‐Actin‐stained WT, *ODF2* KO, and *PLEC* KO SNU‐475 cells grown on fibronectin‐coated coverslips and treated with indicated concentrations of PST for 4 h. For analysis, cell morphology was classified as round (#1), intermediate (#2), and polarized (#3). Nuclei, DAPI. Scalebar, 20 μm. The graph shows the quantification of the percentage of cell shape categories in the indicated SNU‐475 cell cultures. > 100 cells per condition were analyzed; *N* = 4. Data are shown as mean ± SEM. HCC, hepatocellular carcinoma; KO, knockout; *ODF2*, outer dense fiber protein 2; *PLEC*, plectin; PST, plecstatin; WT, wild‐type.

*ODF2* expression was elevated in tumor tissues and correlated with advanced TNM stage and poor overall survival (Fig. 1A–C and Fig. S1C), suggesting a role in tumor progression and metastasis. Based on this observation, and given that PST has been shown to suppress tumor dissemination both *in vitro* and *in vivo* in lung colonization assays using Huh7 and SNU‐475 cells [7], we next investigated whether its anti‐invasive effects are mediated through plectin, ODF2, or both. To address this, we first performed an *in vitro* scratch wound assay to assess the cell migration, a key readout for the cancer dissemination potential. PST induced a dose‐dependent reduction in the migration speed of *ODF2* KO and WT SNU‐475 cells, while having no detectable effect on *PLEC* KO cells (Fig. 3A). At the highest PST concentration, the mean migration speed of WT and *ODF2* KO cells was reduced to that of untreated *PLEC* KO SNU‐475 cells, supporting a plectin‐dependent mechanism. Consistent with this, PST treatment significantly reduced the proportion of WT cells exhibiting polarized morphology, which is indicative of impaired cytoskeletal remodeling and defective protrusion formation (Fig. 3B). The mirrored dose‐dependent response in *ODF2* KO cells and lack of effect in *PLEC* KO cells further implicate plectin, but not ODF2, as the critical mediator of PST‐induced inhibition of cell migration and polarity.

**Fig. 4:**
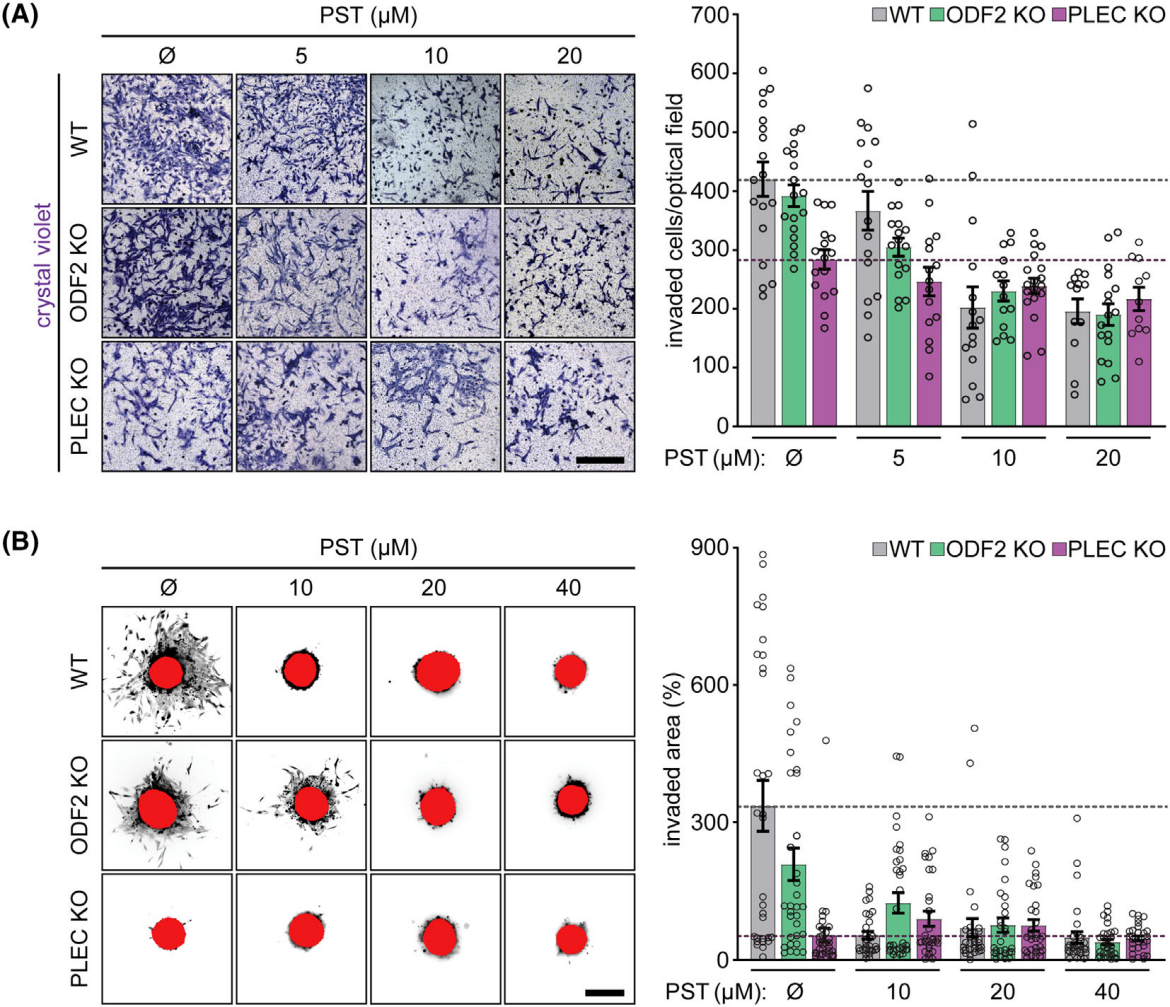
Plectin‐mediated effect of PST treatment on HCC cell invasion potential. (A) Representative images of crystal violet‐stained WT, *ODF2* KO, and *PLEC* KO SNU‐475 cells invading in Matrigel transwell assay, treated with indicated concentrations of PST. Scale bar, 400 μm. The graph shows quantification of the number of invaded cells. Dotted lines, mean control WT (gray) and *PLEC* KO (magenta) invaded cells/area. Data are shown as mean ± SEM; dots, fields of view; *N* = 4. Two‐way ANOVA: treatment (PST concentration, *P* < 0.0001), (WT, *ODF2* KO, *PLEC* KO genotype *P* < 0.001), and interaction treatment × genotype (*P* < 0.05). (B) Representative images of calcein‐stained WT, *ODF2* KO, and *PLEC* KO SNU‐475 spheroids grown for 3 days in collagen mixture, treated with indicated concentrations of PST. Red, initial spheroid area. Scale bar, 400 μm. Graph, invaded area calculated as the percentage of the initial spheroid area from day 0. Dotted lines, mean control WT (gray) and *PLEC* KO (magenta) invaded area. Data are shown as mean ± SEM; dots, individual spheroids; *N* = 4. Two‐way ANOVA: treatment (PST concentration, *P* < 0.0001), (WT, *ODF2* KO, *PLEC* KO genotype *P* < 0.0001), and a treatment × genotype interaction (*P* < 0.01). HCC, hepatocellular carcinoma; KO, knockout; *ODF2*, outer dense fiber protein 2; *PLEC*, plectin; PST, plecstatin; WT, wild‐type.

To determine whether these findings extend to a 3D tumor‐like context, we assessed the impact of PST on invasive behavior using both Matrigel transwell and 3D spheroid invasion assays. In both assays, PST dose‐dependently inhibited the invasion of *ODF2* KO and WT SNU‐475 cells, but did not affect *PLEC* KO cells (Fig. 4A,B), again confirming the requirement of plectin. Importantly, in the 3D spheroid assay, invasion was completely abolished in *PLEC* KO cells and significantly suppressed in WT cells even at the lower PST dose, demonstrating the high potency of plectin‐targeted inhibition of collective tumor invasion in HCC. Interestingly, PST activity in 3D spheroid invasion assays was previously demonstrated in the HCT116 colon carcinoma cell line [6], however, these experiments utilized the less potent PST‐2 variant, which required substantially higher concentrations (> 400 μm). The apparently lower sensitivity of HCT116 cells in the 3D spheroid assay may also reflect their more epithelial‐like phenotype, characterized by cohesive spheroid architecture and consequently reduced invasive outgrowth compared with SNU‐475 cells.

Collectively, these results demonstrate that PST suppresses HCC dissemination primarily by inhibiting the promigratory and pro‐invasive functions of plectin, while ODF2 appears to be dispensable in this context.

### 3.4 PST‐mediated disruption of ODF2‐dependent ciliogenesis

Although ODF2 expression correlates with poor prognosis and increased tumorigenicity in HCC patients, our functional analyses so far do not support a major role for ODF2 in mediating PST antitumorigenic or anti‐invasive effects. To explore whether PST might affect alternative ODF2‐dependent pathways relevant to tumor progression, we performed mass spectrometry‐based shotgun proteomic profiling. Cytosolic and cytoskeleton‐enriched fractions were analyzed from untreated and PST‐treated WT, *ODF2* KO, and *PLEC* KO SNU‐475 cells. For identifying ODF2‐specific PST responses, PST‐treated *ODF2* KO cells were also included.

Using a label‐free quantification strategy, we identified in total 1689, 1281, 1452, and 2186 significantly deregulated proteins in the comparisons WT vs. PST‐treated WT, WT vs. *ODF2* KO, WT vs. *PLEC* KO, and *ODF2* KO vs. PST‐treated *ODF2* KO SNU‐475 cells, respectively (Fig. S5A). Across all groups, cytosolic fractions showed a 2–3‐fold higher number of deregulated proteins compared to cytoskeletal fractions, except in WT vs. *PLEC* KO cells, where cytoskeletal deregulation predominated, consistent with plectin's central role in cytoskeletal organization (Fig. S5A,B). Importantly, global proteomic responses to PST were highly comparable between WT and *ODF2* KO cells, with volcano plots revealing a similar number of deregulated proteins and an overall comparable perturbation pattern (1337 in PST‐treated WT vs. 1462 in PST‐treated *ODF2* KO; Fig. S5A, lower panels). Likewise, PST‐induced effects of comparable magnitude and direction were observed along principal component 1 in both genotypes (Fig. S6A). Notably, *ODF2* KO cells exhibited no alterations in the expression of ABC efflux transporters (Fig. S6B), which are known to confer resistance to anticancer drug therapies [42]. Collectively, these findings provide no evidence for compensatory changes in *ODF2* KO cells that could compromise PST responsiveness.

**Fig. 5:**
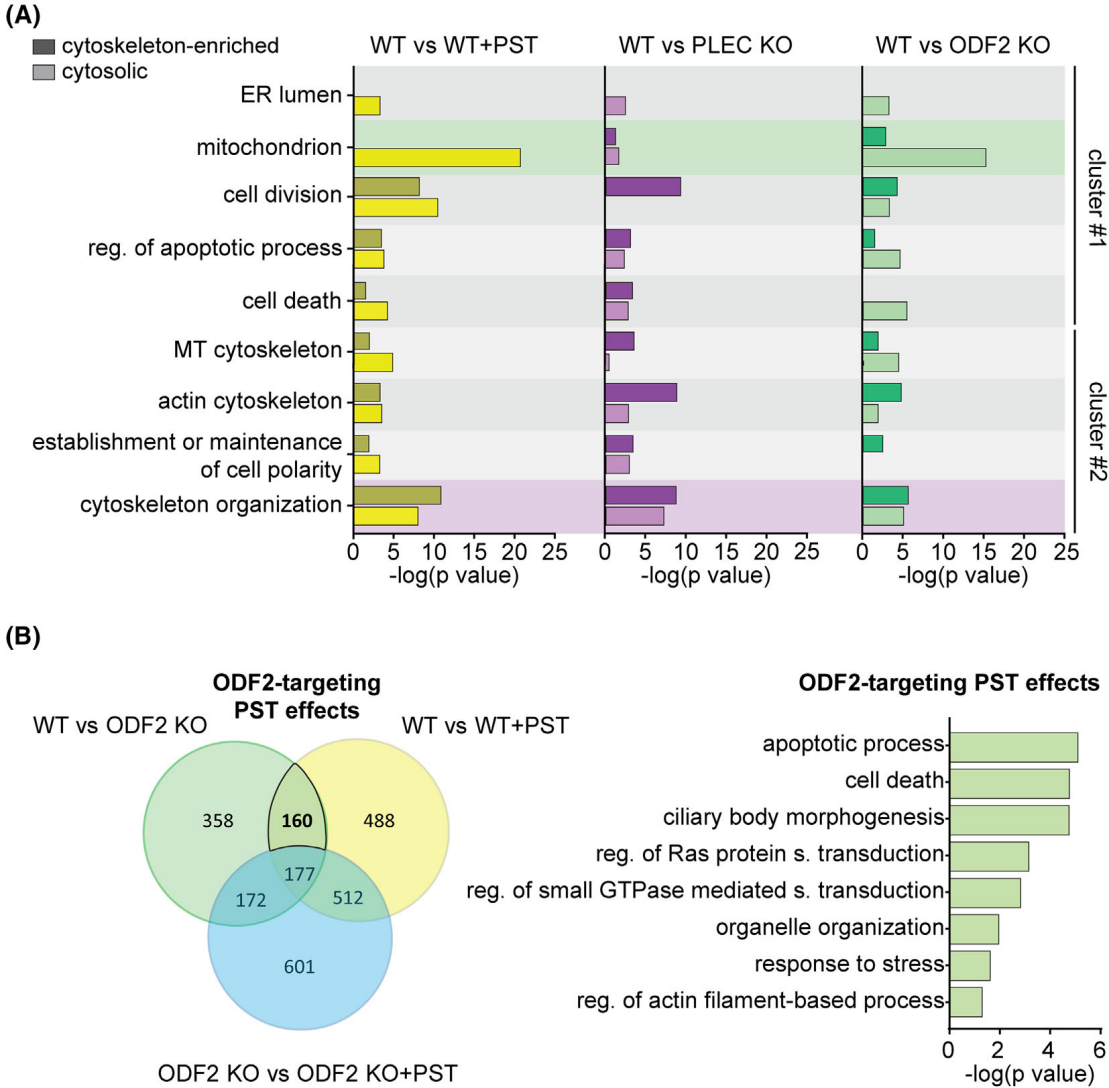
ODF2 as a potential PST effector. MS‐based shotgun proteomics and label‐free quantification (LFQ) of cytosolic and cytoskeletal fractions from SNU‐475 cells. (A) Representative Gene Ontology (GO) enrichment analysis of significantly deregulated proteins. Terms were selected for non‐redundancy, specificity, and significance. The graphs show the −log10 of *P*‐values for enrichment of the different terms in PST‐treated WT (WT + PST), *PLEC* KO, and *ODF2* KO SNU‐475 proteomes. (B) Venn diagram shows the relative proportions of differentially expressed proteins identified by proteomic analyses shared between WT vs. WT + PST, WT vs. *ODF2* KO, and *ODF2* KO vs. *ODF2* KO + PST SNU‐475 cells. Note that 160 proteins are shared between WT vs. *ODF2* KO and WT vs. WT + PST comparisons, but not with *ODF2* KO vs. *ODF2* KO + PST. These proteins represent the specific ODF2‐targeting PST signature, with corresponding GO enrichment shown on the right (*x*‐axis, −log10 of *P*‐values). Terms were selected for non‐redundancy, specificity, and significance. Two‐sided Student's *t*‐test with multiple testing correction: *FDR* < 0.05; *S*_0_ = 0.1; *N* = 4. KO, knockout; MS, mass spectrometry; *ODF2*, outer dense fiber protein 2; *PLEC*, plectin; PST, plecstatin; WT, wild‐type.

Gene Ontology (GO) enrichment analysis of untreated WT vs. PST‐treated WT SNU‐475 cells based on shared cluster analysis with WT vs. *PLEC* KO and WT vs. *ODF2* KO revealed two major clusters of biological processes: (1) processes related to cell division, apoptosis, cell death, as well as endoplasmic reticulum (ER) and mitochondrial function, and (2) cytoskeletal organization, microtubule and actin cytoskeleton, and establishment or maintenance of cell polarity (Fig. 5A). As anticipated, *PLEC* KO cells exhibited stronger enrichment in cytoskeletal processes (cluster #2), while *ODF2* KO cells showed a more pronounced response in mitochondrial and apoptotic pathways (cluster #1), implicating distinct stress response axes.

Analysis of overlapping proteins between respective comparisons (WT vs. *ODF2* KO, WT vs. PST‐treated WT, and *ODF2* KO vs. PST‐treated *ODF2* KO) revealed that a large number of deregulated proteins (*N* = 512) were shared between PST‐treated *ODF2* KO and WT cells (Fig. 5B). This finding is consistent with our previous results indicating that PST responsiveness is largely preserved in *ODF2*‐deficient cells. To further assess potential compensatory effects in *ODF2* KO cells, we compared PST responses in WT and *ODF2* KO backgrounds with respect to plectin expression, cytoskeletal remodeling, and redox homeostasis [6, 43]. PST‐treated WT and *ODF2* KO cells displayed similar plectin levels and comparable response patterns involving cytoskeleton‐associated proteins, including vimentin (VIM), thymosin β‐4 (TMSB4X), desmoplakin (DSP), integrins (ITGB1 and ITGB3), and ADAM10, as well as redox‐related proteins including HMOX1, SRXN1, NQO1, and OSGIN1 (clustering of dark and light‐yellow shades in Fig. S6C). Altogether, these results indicate that PST‐induced perturbations retain key features of PST specificity, irrespective of ODF2 genotype, in SNU‐475 cells.

To dissect the ODF2‐specific component of the PST response, we performed GO enrichment analysis on deregulated proteins shared between *ODF2* KO versus WT and PST‐treated WT versus WT, while excluding proteins altered in PST‐treated *ODF2* KO cells. Among the top‐enriched terms, we identified ODF2‐targeted subsets associated with ‘ciliary body morphogenesis,’ as well as ‘apoptotic processes’ and ‘cell death’ (Fig. 5B), the latter likely reflecting activation of the Integrated Stress Response (ISR) upon PST treatment [44]. The enrichment of ciliary processes is consistent with the established role of the ODF2 isoform cenexin (isoform 9) in ciliogenesis [20, 21].

**Fig. 6:**
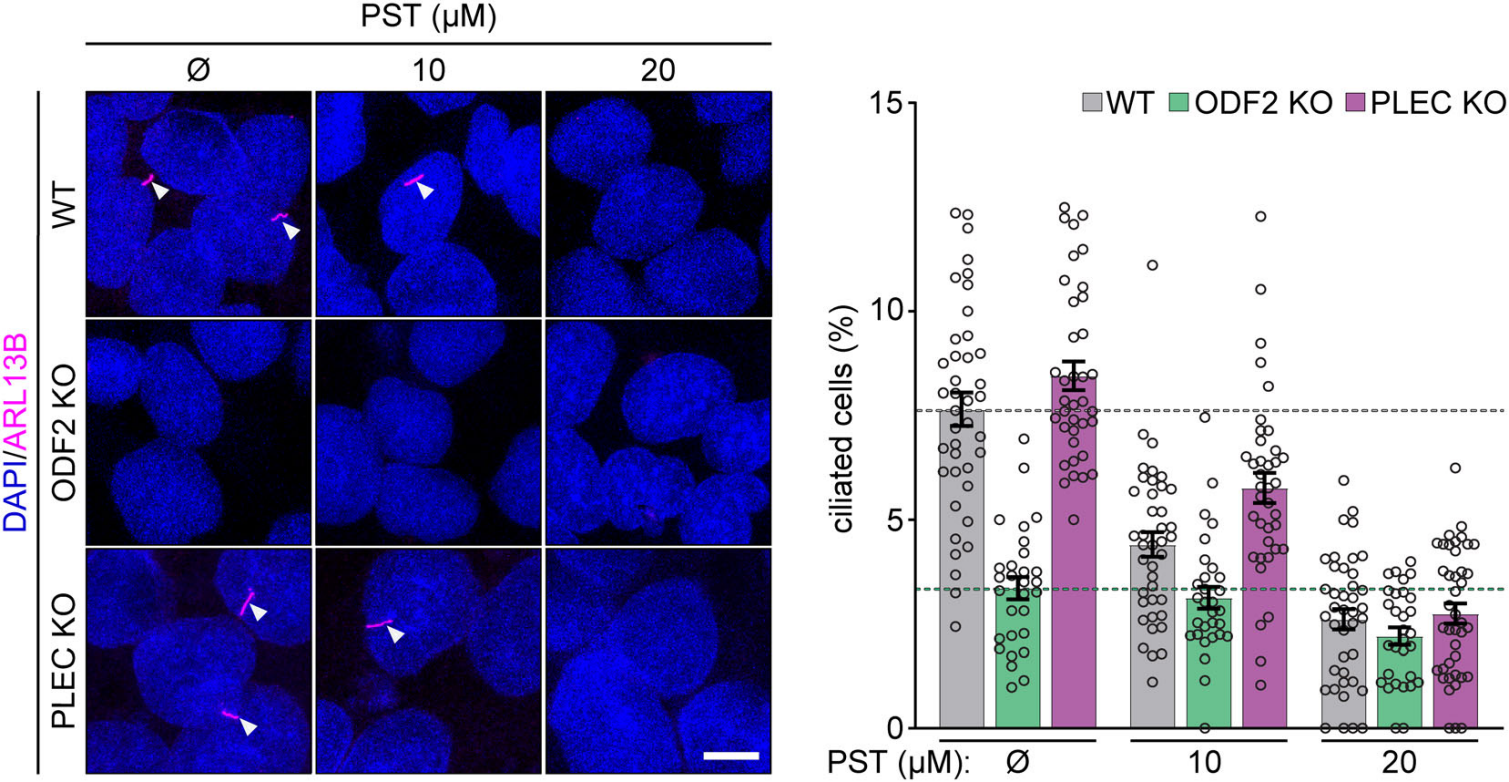
Effects of PST treatment on ciliogenesis are ODF2‐mediated. Representative confocal micrographs of WT, *ODF2* KO, and *PLEC* KO SNU‐475 cells, treated with indicated concentrations of PST during 2 days of serum starvation and immunostained for ARL13B (magenta). Nuclei, DAPI. Arrowheads, cilia. Scale bar, 10 μm. The graph shows the quantification of the percentage of ciliated cells. Dotted lines, mean values for control WT (gray) and *ODF2* KO (green) ciliated cells. Data are shown as mean ± SEM; dots, fields of view; *N* = 4. Two‐way ANOVA: (PST concentration, treatment *P* < 0.0001), genotype (WT, *ODF2* KO, *PLEC* KO *P* < 0.0001), and a treatment × genotype interaction (*P* < 0.0001). KO, knockout; *ODF2*, outer dense fiber protein 2; *PLEC*, plectin; PST, plecstatin; WT, wild‐type.

To validate this cilia‐associated signature, we assessed the impact of PST on primary cilium formation. Fully confluent, serum‐starved WT, *PLEC* KO, and *ODF2* KO SNU‐475 cells were treated with PST, and ciliogenesis was evaluated by quantifying cells positive for the ciliary marker, the adenosine diphosphate‐ribosylation factor‐like protein 13B (ARL13B). As expected, *ODF2* KO cells exhibited significantly reduced baseline ciliogenesis compared to WT and *PLEC* KO cells (Fig. 6). PST treatment resulted in a dose‐dependent reduction of ciliated cells in WT and *PLEC* KO populations, while ODF2 KO cells showed no further reduction, confirming that PST inhibits ciliogenesis in an ODF2‐dependent manner.

Taken together, these data reveal that although ODF2 is dispensable for PST's anticancer efficacy, it contributes to a distinct PST‐targetable axis related to ciliogenesis and stress responses that may be of interest in the context of sensory ciliopathies [45]. This highlights a secondary, nonessential pathway of PST action, with implications for understanding off‐target or tissue‐specific effects of cytoskeleton‐interacting metallodrugs.

### 3.5 PST‐induced integrated stress response linked to apoptosis depends on both plectin and ODF2

**Fig. 7:**
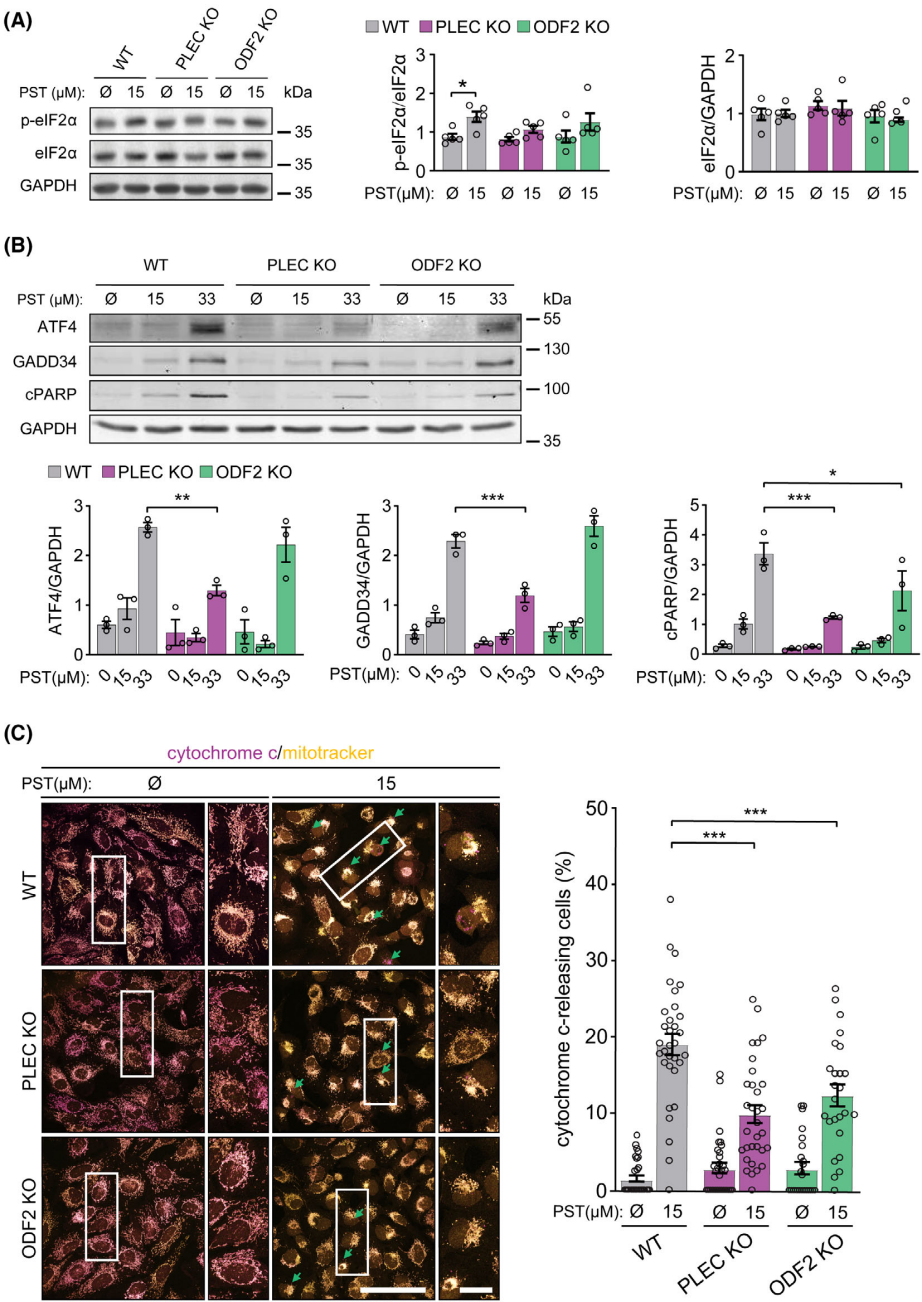
Plectin‐ and ODF2‐mediated effect of PST treatment on ISR in HCC cells. (A) Quantification of phospho‐Ser51‐eIF2α (p‐eIF2α) and eIF2α by immunoblotting in WT, *ODF2* KO, and *PLEC* KO SNU‐475 cells treated with 15 μm PST for 16 h. GAPDH, loading control. Graphs show relative band intensities normalized to GAPDH or non‐phosphorylated eIF2α. MWs: eIF2α, 38 kDa; GAPDH, 36 kDa. Data are shown as mean ± SEM; dots, individual experiments; *N* = 5. Two‐way ANOVA, Dunn‐Sidak multiple comparison test, *P < 0.05. (B) Quantification of ATF4, GADD34, and cleaved PARP by immunoblotting in WT, *ODF2* KO, and *PLEC* KO SNU‐475 cells treated with 15 and 33 μm PST for 16 h. GAPDH, loading control. Graphs show relative band intensities normalized to GAPDH. MWs: ATF4, 49 kDa; GADD34, 110 kDa; cPARP, 89 kDa; GAPDH, 36 kDa. Data are shown as mean ± SEM; dots, individual experiments; *N* = 3. Two‐way ANOVA, Dunn‐Sidak multiple comparison test, *P < 0.05, **P < 0.01, ***P < 0.001. (C) Representative confocal images of WT, *PLEC* KO, and *ODF2* KO SNU‐475 cells stained for mitochondria (mitotracker, yellow) and cytochrome c (magenta). Arrows, cytochrome c release. Boxed areas, 2× images. Scale bar, 100 and 25 μm (boxed areas). The graph shows the quantification of the percentage of cytochrome c‐releasing cells. Data are shown as mean ± SEM; dots, fields of view; *N* = 4. Two‐way ANOVA, Dunn‐Sidak multiple comparison test, ***P < 0.001. HCC, hepatocellular carcinoma; KO, knockout; MWs, Molecular weights; *ODF2*, outer dense fiber protein 2; *PLEC*, plectin; PST, plecstatin; WT, wild‐type.

PST has previously been shown to induce ISR, mitochondrial stress, and apoptosis [44]. Given the lack of ODF2 involvement in PST‐mediated migration and invasion, and our proteomic indications of an ODF2‐dependent component within the PST‐induced ISR pathway, we next examined ISR activation in this context. To assess ISR status, we analyzed the phosphorylation of eIF2α at serine 51 in PST‐treated and untreated WT, PLEC KO, and ODF2 KO SNU‐475 cells, as this modification represents a hallmark initiating event of ISR activation [46]. PST treatment markedly increased eIF2α phosphorylation in WT cells, consistent with robust ISR induction (Fig. 7A). In contrast, both *PLEC* KO and *ODF2* KO cells displayed substantially attenuated responses, with eIF2α phosphorylation failing to reach the levels observed in WT cells (Fig. 7A).

We next examined whether PST treatment affects the levels of Activating Transcription Factor 4 (ATF4) and GADD34, key downstream effectors of the ISR (reviewed in [46]). ATF4 is a transcription factor whose translation selectively increases during ISR activation, regulating genes involved in amino acid biosynthesis, antioxidant defense, and cell survival or apoptosis. *GADD34* expression is induced by ATF4, forming a negative feedback loop that promotes dephosphorylation of eIF2α and thereby modulates ISR intensity. PST treatment significantly increased the levels of both ATF4 and GADD34 in WT cells (Fig. 7B). Consistent with the effects observed on eIF2α phosphorylation, PST‐induced upregulation of ATF4 and GADD34 was attenuated in both *PLEC* KO and *ODF2* KO cells, reaching statistical significance in *PLEC* KO cells (Fig. 7B). Together, these results indicate that both plectin and ODF2 contribute to efficient PST‐induced ISR activation (Fig. 7B).

To strengthen the clinical relevance of the proposed ISR mechanism, we analyzed publicly available HCC patient datasets and performed correlation analyses between plectin and ODF2 expression levels and established ISR‐associated gene signatures. Higher expression of both plectin and ODF2 correlated positively with multiple ISR‐related gene signatures (Fig. S7). Notably, ODF2 displayed a stronger and more consistent association with ISR than plectin. Additional correlations were observed with specific clinicopathological parameters and molecular classifications, including Hoshida subgroup S1, which is characterized by elevated TGFβ‐signaling and metabolic stress (Fig. S7 and [47, 48]). Collectively, these findings provide strong clinical support for the relevance of the proposed ISR mechanism.

Activation of ISR represents a key step in cancer progression, promoting either adaptive survival or, when sustained, selective apoptotic cell death [46]. To determine whether PST induces apoptosis in SNU‐475 cells, we assessed levels of cleaved poly(ADP‐ribose) polymerase (cPARP), a hallmark of apoptosis required for chromatin decondensation [49]. To evaluate potential dose‐dependent effects, we also included a higher PST concentration of 33 μm. In parallel, we examined mitochondrial cytochrome c release, as this event is known to accompany PST‐induced mitochondrial stress and ISR‐mediated apoptosis [43, 44].

As expected, PST treatment markedly increased cPARP levels in WT cells (Fig. 7B), confirming PST‐induced apoptotic activity. PST‐mediated PARP cleavage required both plectin and ODF2, as cPARP induction was significantly reduced in PST‐treated *PLEC* KO and *ODF2* KO cells (Fig. 7B). Consistently, in WT cells, PST treatment led to a robust release of cytochrome c, evident by a substantial increase in the fraction of cells exhibiting non‐overlapping cytochrome c and mitochondrial signals (Fig. 7C). In contrast, both *PLEC* KO and *ODF2* KO cells exhibited a significantly milder response, approximately 50% of the release observed in WT cells, mirroring reduced cPARP levels. Collectively, these results suggest that plectin and ODF2 cooperate in regulating PST‐induced mitochondrial stress‐linked apoptosis, likely acting as complementary modulators of stress signaling. While individually dispensable for PST antitumor and anti‐invasive effects, both proteins appear to support the full pro‐apoptotic output of PST.

## 4 Discussion

Deciphering the mechanism of action and target specificity of metal‐based drug candidates remains a key challenge, particularly given the phenotypic nature of their discovery pipelines [50]. While multitargeted therapies offer advantages in combating resistance, identifying the principal targets and affected pathways is essential to minimize off‐target toxicity and improve clinical translation [51]. In this study, we dissected the anticancer mechanism of the ruthenium‐based prodrug PST, with a particular focus on its selectivity for plectin and ODF2, previously identified as putative interactors by proteomic profiling [6].

Our study establishes that the major antitumorigenic and anti‐invasive effects of PST in HCC cells are mediated through plectin, not ODF2. Using CRISPR/Cas9‐based gene editing, we demonstrated that plectin deficiency abrogates PST‐induced suppression of anchorage‐independent growth and 2D/3D migration, while *ODF2* KO retains sensitivity. These findings resolve prior uncertainties regarding the contribution of ODF2 and highlight plectin as the principal functional target responsible for PST therapeutic effects in hepatocellular carcinoma. At the same time, our data reveal a previously unrecognized role for ODF2 in promoting tumorigenesis and collective HCC cell invasion independent of its interaction with PST. Moreover, we identify a shared contribution of both plectin and ODF2 to PST‐triggered activation of the ISR [46], suggesting a partial convergence of stress‐related pathways downstream of PST exposure.

Target identification for metal‐based compounds such as PST is complicated by the dynamic nature of prodrug activation, ligand exchange, and intracellular speciation under tumor‐specific redox conditions [51, 52]. PST is administered in a metal‐halogen prodrug form and undergoes intracellular hydrolysis, yielding a species that preferentially binds selective targets (see schematics in Fig. S1A). While MS‐based profiling enables detection of protein interactors in native environments, functional validation remains essential. Here, we provide direct evidence that plectin and ODF2 are *bona fide* PST interactors but play distinct roles in mediating downstream effects. Furthermore, PST efficacy has previously been demonstrated in another HCC cell line, Huh7, which possesses a distinct genetic background and phenotypic profile (Cellosaurus [41, 53]) and which we show to belong to a different Dr Boyault's subgroup (Table S2). Moreover, the expression of plectin, the primary target of PST, is not associated with a specific mutation that is frequently found in HCC (Fig. S3B). We therefore conclude that PST's effects are not confined to a single mutational or differentiation context but are conserved across genetically and phenotypically diverse HCC models.

The lack of any detectable response to PST in *PLEC* KO cells, contrasted with a clear dose‐dependent response of *ODF2* KO SNU‐475 cells, in tumorigenic soft agar assay, as well as 2D and 3D migration assays, allows us to conclude that PST major anticancer effects on growth and invasiveness are mediated through plectin and not ODF2. In contrast, proteomic profiling of the PST‐response signature associated with ODF2 revealed ‘ciliary body morphogenesis’ as the top‐enriched biological process. This functional association was validated by our observation that, unlike *PLEC* KO cells, *ODF2* KO SNU‐475 cells displayed markedly reduced ciliation and were unresponsive to PST in ciliogenesis. While PST inhibition of ODF2‐dependent ciliogenesis may hold relevance in specific oncogenic contexts, this effect does not appear to play a major role in the drug's core anticancer activities, which are clearly governed by its action on plectin. Furthermore, in HCC and other somatic cells, the predominant ODF2 isoform, cenexin‐1 (ODF2 isoform 9), localizes to the basal body, where it plays a central role in primary cilia assembly [20, 21]. Primary cilia function predominantly as sensory organelles and signaling hubs rather than motility structures, and their dysfunction is implicated in disease contexts such as sensory ciliopathies [45].

We further show that PST activates the ISR in HCC cells, consistent with previous findings in colon cancer models, suggesting that ISR induction by PST represents a broader, tissue‐independent mechanism of action [6, 43, 44]. This response involves phosphorylation of eukaryotic initiation factor 2α (eIF2α), a hallmark initiating event of ISR signaling, as well as its key effector targets ATF4 and GADD34 [46], which we found markedly attenuated in both *PLEC* KO and *ODF2* KO SNU‐475 cells. Furthermore, strong correlation of both plectin and ODF2 expression with multiple ISR‐related gene signatures derived from HCC patient datasets highlights the clinical relevance of the PST‐plectin‐ODF2‐ISR axis. These data indicate that both plectin and ODF2 contribute to full ISR induction upon PST exposure. However, additional pro‐apoptotic outputs of PST, particularly those involving mitochondrial stress and cytochrome c release, may involve further, low‐affinity or transient protein interactions [43, 52]. Such early interactions are characteristic of the ISR's broad sensory capacity and may serve to prime cells for apoptosis when stress cannot be resolved. Although short‐lived, these interactions could help initiate mitochondrial stress responses or amplify downstream apoptotic signaling. Following prodrug activation via hydrolysis (Fig. S1A), PST preferentially engages more stable, high‐affinity targets such as histidine‐rich pockets within plectin and potentially ODF2, thereby refining its action toward protein‐selective cytotoxicity [6, 43]. Importantly, the relatively rapid activation kinetics of PST (~ 50 min) likely limit systemic off‐target effects, reinforcing its potential as a targeted therapeutic agent [6].

Compared to classical platinum‐based agents such as cisplatin or carboplatin, which act through persistent DNA damage and are often limited by toxicity and resistance [54, 55, 56], PST offers a protein‐targeting mechanism that bypasses DNA binding. The absence of DNA‐binding properties in PST may allow for fewer side effects and higher tolerability, making it a unique protein‐binding metallodrug candidate against HCC [6]. Consistent with this, we could show previously in preclinical models that oral administration of PST is well‐tolerated over extended periods [6, 7]. This is in line with findings of a prodrug activation mechanism, which is thought to occur inside the cells, preferentially in cancer cells [43, 52]. Moreover, free drug was not detected in serum and was found mainly bound to albumin [57], altogether making it a highly suitable candidate drug to be translated to a clinical setting.

Mechanistically, prior studies [6, 7, 9] and our current proteome analyses comparing PST‐treated WT and *PLEC* KO SNU‐475 cells support the notion that PST exerts its primary anticancer effects by disrupting plectin‐mediated cytoskeletal organization (see Graphical Abstract). Our quantitative morphometric analysis presented here reveals that PST exerts its anti‐migratory effects through an inhibitory effect on plectin‐dependent cell polarization. This aligns with GO enrichment results identifying ‘establishment of cell polarity’ as a top deregulated pathway in both PST‐treated WT and *PLEC* KO cells. These findings are consistent with earlier observations that cytoskeletal alterations in *PLEC* KO cells phenocopy those seen in PST‐treated WT cells [7, 9]. Importantly, these cellular effects are recapitulated *in vivo*, where pharmacological plectin inhibition via PST mirrors the anticancer phenotype of genetic plectin ablation in liver cancer models [7]. At the mechanistic core, plectin's role as a cytoskeletal crosslinker is essential not only for cell migration but also for anchorage‐dependent growth. By anchoring intermediate filaments to focal adhesions and regulating adhesome‐dependent mechanotransduction, plectin orchestrates both structural integrity and proliferative signaling [7, 58, 59].

Taken together, our findings underscore the value of mechanistic dissection in the development of protein‐targeting metallodrugs. While our study builds on phenotypic responses to PST, we extend this approach by applying genetic dissection to delineate the molecular specificity of action. By functionally separating the contributions of plectin and ODF2, we refine our understanding of PST's protein‐selective effects in HCC cells. These insights pave the way for leveraging cytoskeletal scaffolds as precision‐entry points in drug design, expanding the scope of rational therapies beyond traditional nucleic acid‐directed agents.

## 5 Conclusion

This study demonstrates that the potent anticancer effects of PST on tumor growth and invasiveness are mediated by its selective targeting of plectin, with no evidence for a major contribution from ODF2. PST strongly and dose‐dependently suppresses anchorage‐independent growth in wild‐type and *ODF2*‐deficient SNU‐475 hepatocellular carcinoma cells, while *PLEC*‐deficient cells remain unresponsive, underscoring the drug's specificity for plectin. Similarly, PST significantly reduces cancer cell migration and invasion in both 2D and 3D models in a plectin‐dependent manner. While an ODF2‐related PST signature linked to ciliary pathways was identified, it appears peripheral to the drug's primary anticancer activity. Both plectin and ODF2 contribute to PST‐induced activation of the integrated stress response, which modulates cancer cell survival and apoptosis. PST, plecstatin.

## Conflict of interest

The authors declare no conflict of interest.

## Author contributions

ZO designed and performed experiments, formal analysis, and figure assembly and wrote the original draft. JK designed and performed experiments and figure assembly during revision, provided intellectual input, performed formal analysis, and contributed to the writing of the manuscript. PB, YB, AB, CG, and SMM‐M performed proteomics, including analysis and data curation. MP and LF analyzed proteomic and patient data, including data curation. LF conducted additional analyses of patient datasets for ISR signatures, HCC subtype classification, and *ODF2/PLEC* correlations with gene mutations during revision. SO‐M provided intellectual input and, together with MG, co‐wrote the manuscript. MG designed and conceived the project, supervised its execution, and provided resources. All authors approved, reviewed, and edited the manuscript.

## Supporting information


**Fig. S1.** Plectin and ODF2 targeting by PST and ODF2 expression in hepatocellular carcinoma.
**Fig. S2.** Gene expression patterns of both plectin and ODF2 across the entire HCC tumor microenvironment.
**Fig. S3.** Association of plectin and ODF2 expression with molecular subclasses of HCC and mutations.
**Fig. S4.** Verification of plectin and ODF2 gene depletion in SNU‐475 cell lines and the effect of PST treatment on colony size.
**Fig. S5.** Analysis of proteomic signatures of PST treatment, plectin ablation, and ODF2 ablation in SNU‐475 cells.
**Fig. S6.** Absence of compensatory effects in *ODF2* KO SNU‐475 cells.
**Fig. S7.** Plectin and ODF2 ISR‐related signature in HCC patients.
**Table S1.** Primary and secondary antibodies used in this study.
**Table S2.** The classification of 23 HCC cell lines using RNA expression‐based nearest template prediction (NTP) into Dr Boyault's molecular subgroups (G1–G6) of HCC.

## Data Availability

Proteomic data were submitted to the ProteomeXchange Consortium (https://proteomecentral.proteomexchange.org/) and are available in the PRIDE partner repository [60] with identifier PXD060086 (https://www.ebi.ac.uk/pride/archive/projects/PXD060086).
