# Supplementary material for: Plecstatin inhibits hepatocellular carcinoma tumorigenesis and invasion through cytolinker plectin
Source: Mol Oncol. 2025 Dec 30;20(6):1453–72. doi: 10.1002/1878-0261.70186 (PMC13238617; doi:10.1002/1878-0261.70186)
Supplement: Supplementary file 1 — Fig. S1. Plectin and ODF2 targeting by PST and ODF2 expression in hepatocellular carcinoma. Fig. S2. Gene expression patterns of both plectin and ODF2 across the entire HCC tumor microenvironment. Fig. S3. Association of plectin and ODF2 expression with molecular subclasses of HCC and mutations. Fig. S4. Verification of plectin and ODF2 gene depletion in SNU‐475 cell lines and the effect of PST treatment on colony size. Fig. S5. Analysis of proteomic signatures of PST treatment, plectin ablation, and ODF2 ablation in SNU‐475 cells. Fig. S6. Absence of compensatory effects in ODF2 KO SNU‐475 cells. Fig. S7. Plectin and ODF2 ISR‐related signature in HCC patients. Table S1. Primary and secondary antibodies used in this study. Table S2. The classification of 23 HCC cell lines using RNA expression‐based nearest template prediction (NTP) into Dr Boyault's molecular subgroups (G1–G6) of HCC. [file MOL2-20-1453-s001.zip › mol270186-sup-0001-Tables.docx]

1. Supplementary Tables

| **Primary antibodies** | | | | | |
| --- | --- | --- | --- | --- | --- |
| **Antigen** | **Clone** | **Manufacturer** | **Cat. No.** | **Application** | **Dilution** |
| rabbit anti-ARL13B | polyclonal | Proteintech | 17711-1-AP | immunofluorescence | 1:200 |
| rabbit anti-ATF-4 | D4B8 | Cell Signaling | 11815 | WB | 1:100 |
| rabbit anti-cytochrome c | 37BA11 | Abcam | 45-6100 | immunofluorescence | 1:200 |
| mouse anti-eIF2α | L57A5 | Cell Signaling | 2103 | WB | 1:500 |
| mouse anti-peIF2α (Ser51) | D9G8 | Cell Signaling | 3398 | WB | 1:500 |
| rabbit anti-GAPDH | polyclonal | Sigma | G9545 | WB | 1:20.000 |
| rabbit anti-GADD34 | E3S6N | Cell Signaling | 41222 | WB | 1:1000 |
| rabbit anti-ODF2 | polyclonal | Abcam | ab43840 | WB | 1:500 |
|  |  |  |  | immunofluorescence | 1:100 |
| rabbit anti-cleaved PARP (Asp214) | D64E10 | Cell Signaling | 5625 | WB | 1:1000 |
| guinea pig anti-plectin | polyclonal | Progen | GP21 | WB | 1:1000 |
|  |  |  |  | immunofluorescence | 1:250 |
| **Secondary antibodies** | | | | | |
| **Name** | **Manufacturer** | **Cat. No.** | **Application** | **Dilution** |  |
| anti-mouse AF-488 | Jackson ImmunoResearch | 715-545-150 | immunofluorescence | 1:500 |  |
| anti-rabbit AF-488 | Jackson ImmunoResearch | 711-545-152 | immunofluorescence | 1:500 |  |
| anti-rabbit AF-594 | Life Tech | A11037 | immunofluorescence | 1:1000 |  |
| anti-rabbit AF-647 | Jackson ImmunoResearch |  | immunofluorescence | 1:500 |  |
| anti-guinea pig AF-488 | Jackson ImmunoResearch | 706-545-148 | immunofluorescence | 1:500 |  |
| anti-mouse IgG IRDye® 680RD | Licor | 926-68072 | WB | 1:20,000 |  |
| anti-mouse IRDye® 800CW | Licor | 926-32212 | WB | 1:20,000 |  |
| anti-rabbit IRDye® 680RD | Licor | 926-68073 | WB | 1:20,000 |  |
| anti-rabbit IgG IRDye® 800CW | Licor | 926-32213 | WB | 1:20,000 |  |
| anti-guinea pig IRDye® 680RD | Licor | 926-68077 | WB | 1:20,000 |  |

**Table S1.** Primary and secondary antibodies used in this study.

| **Cell line** | **Prediction** | **distance G1** | **distance G2** | **distance G3** | **distance G4** | **distance G5** | **distance G6** | **p value** | **FDR** |
| --- | --- | --- | --- | --- | --- | --- | --- | --- | --- |
| HEP3B217 | G1 | 0,5208 | 0,6919 | 0,6835 | 0,7202 | 0,6983 | 0,7269 | 0,0010 | 0,0021 |
| JHH7 | G3 | 0,6052 | 0,6960 | 0,5918 | 0,7048 | 0,6873 | 0,6930 | 0,0010 | 0,0021 |
| SNU878 | G6 | 0,7862 | 0,7096 | 0,6883 | 0,6885 | 0,7221 | 0,6665 | 0,0010 | 0,0021 |
| SNU739 | G1 | 0,6274 | 0,7030 | 0,6810 | 0,6856 | 0,6888 | 0,6592 | 0,0440 | 0,0674 |
| SKHEP1 | G5 | 0,8096 | 0,7207 | 0,7630 | 0,7150 | 0,7089 | 0,7538 | 0,2458 | 0,2975 |
| SNU449 | G1 | 0,6904 | 0,7211 | 0,7317 | 0,7348 | 0,6912 | 0,6980 | 0,2208 | 0,2821 |
| JHH4 | G5 | 0,7519 | 0,7308 | 0,7543 | 0,7140 | 0,7042 | 0,7316 | 0,3646 | 0,4193 |
| HLF | G3 | 0,7291 | 0,7083 | 0,5567 | 0,7251 | 0,7098 | 0,7002 | 0,0010 | 0,0021 |
| SNU182 | G6 | 0,6854 | 0,7023 | 0,7142 | 0,7056 | 0,7045 | 0,6786 | 0,9271 | 0,9271 |
| SNU761 | G4 | 0,7568 | 0,7155 | 0,8713 | 0,7019 | 0,7103 | 0,7212 | 0,1758 | 0,2379 |
| JHH2 | G2 | 0,7314 | 0,6840 | 0,7821 | 0,7103 | 0,7277 | 0,7048 | 0,0290 | 0,0476 |
| JHH5 | G1 | 0,6938 | 0,6996 | 0,7490 | 0,7044 | 0,6970 | 0,7404 | 0,0609 | 0,0876 |
| **HUH7** | **G1** | 0,6543 | 0,7097 | 0,7274 | 0,6806 | 0,6953 | 0,7055 | 0,0010 | 0,0021 |
| SNU886 | G3 | 0,6989 | 0,7232 | 0,6131 | 0,6898 | 0,7177 | 0,6798 | 0,0010 | 0,0021 |
| PLCPRF5 | G2 | 0,7405 | 0,6792 | 0,6932 | 0,7365 | 0,7158 | 0,7189 | 0,0220 | 0,0389 |
| SNU423 | G3 | 0,7119 | 0,7063 | 0,6273 | 0,7199 | 0,7077 | 0,6905 | 0,0010 | 0,0021 |
| SNU398 | G3 | 0,6120 | 0,6937 | 0,5766 | 0,7219 | 0,7098 | 0,6887 | 0,0010 | 0,0021 |
| HUH1 | G4 | 0,8289 | 0,7183 | 0,7874 | 0,6892 | 0,7195 | 0,7521 | 0,0040 | 0,0077 |
| JHH1 | G5 | 0,7250 | 0,7126 | 0,8131 | 0,7122 | 0,7093 | 0,7276 | 0,6653 | 0,7144 |
| LI7 | G3 | 0,7188 | 0,7019 | 0,6683 | 0,7034 | 0,7026 | 0,7124 | 0,0010 | 0,0021 |
| SNU387 | G3 | 0,7153 | 0,7222 | 0,6476 | 0,6975 | 0,7324 | 0,7053 | 0,0010 | 0,0021 |
| JHH6 | G5 | 0,7270 | 0,7413 | 0,7338 | 0,7012 | 0,6986 | 0,7048 | 0,6833 | 0,7144 |
| **SNU475** | **G3** | 0,7117 | 0,6800 | 0,6537 | 0,7030 | 0,6997 | 0,6916 | 0,0010 | 0,0021 |

**Table S2.** The classification of 23 HCC cell lines using RNA expression-based nearest template prediction (NTP) into Dr. Boyault’s molecular subgroups (G1-G6) of HCC.

# Supplementary figure legends

**Fig. S1**. Plectin and ODF2 targeting by PST and ODF2 expression in hepatocellular carcinoma. A) A scheme depicting PST’S mode of action. PST is S, N-bidentate pyridinecarbothioamide ruthenium(arene) complex delivered in a prodrug form that requires hydrolysis of the metal-chloride bond for activation. Activation (reactive state) in turn induces ligand switching by promoting reaction with nucleophilic molecules, preferentially histidine in plectin and ODF2. Due to binding to serum proteins and likely due to the high concentration of chloride ions in plasma, before entering the cells, PST is quite inert in plasma and therefore exerts low toxicity. (B) Schematic summary of identification of plectin (PLEC) and ODF2 as major plecstatin interactors, published in[6]. (C) Meta-analysis of differential *ODF2* mRNA expression in non-tumor (NT) liver and hepatocellular carcinoma (HCC) patients. Brown squares indicate the standardized mean difference (SMD) and 95% confidence interval of individual datasets. The black diamond shows the mean and 95% confidence interval for the combined SMD, while the whiskers indicate the 95% prediction interval. *ODF2*, outer dense fiber protein 2; *PLEC*, plectin; PST, plecstatin.

**Fig. S2.** Gene expression patterns of both plectin and ODF2 across the entire HCC tumor microenvironment. This analysis used the Liver Single Cell Atlas from the University of Hong Kong (<https://patholiver.hku.hk/liverp/>)[37], which encompasses datasets with accession numbers [GSE112271](https://www.ncbi.nlm.nih.gov/geo/query/acc.cgi?acc=GSE112271), [GSE149614](https://www.ncbi.nlm.nih.gov/geo/query/acc.cgi?acc=GSE149614), [GSE156625,](https://www.ncbi.nlm.nih.gov/geo/query/acc.cgi?acc=GSE156625) and PRJNA932937 from 24 patients, as described in[37]. DC, dendritic cell; HCC, hepatocellular carcinoma; NK cell, natural killer cell; *ODF2*, outer dense fiber protein 2; *PLEC*, plectin; PST, plecstatin.

**Fig. S3.** Association of plectin and ODF2 expression with molecular subclasses of HCC and mutations. (A) mRNA expression of plectin (*PLEC*) and *ODF2* in the indicated molecular subclasses of DepMap RNA expression HCC. The HCC subclasses were predicted using the Nearest Template Prediction method. The numbers of included patients per cohort are indicated in the graph. Points, individual patient tissue samples. Mann−Whitney *U* test. (B) Association of plectin and *ODF2* mRNA expression with the most common mutations in HCC. Points, individual patient tissue samples. The numbers of included patients per cohort are indicated in the graph. Points, individual patient tissue samples. Mann−Whitney *U* test, Holm Bonferroni correction. HCC, hepatocellular carcinoma; *ODF2*, outer dense fiber protein 2; *PLEC*, plectin.

**Fig. S4.** Verification of plectin and ODF2 gene depletion in SNU-475 cell lines and the effect of PST treatment on colony size. Schematic of CRISPR/Cas9-based strategy for the generation of *PLEC* KO and *ODF2* KO SNU-475 lines. To generate *PLEC* KO, exon 6 of plectin was targeted by the single guide RNA (sgRNA) as previously described[7]. To generate *ODF2* KO, exon 6 of ODF2 was targeted by sgRNA, with color lines depicting sgRNA sequences. Generated mutations are indicated in red. For details, see the Materials and Methods section. (B) Representative immunoblots for plectin and ODF2 in WT, *PLEC* KO, and *ODF2* KO SNU-475 cell lines. GAPDH, loading control. (C) Representative images of WT, *PLEC* KO, and *ODF2* KO SNU-475 cells immunolabeled for plectin (magenta) and ODF2 (green). Nuclei, DAPI (blue). Boxed areas, 3.5x images. Scale bar, 50 µm and 14 μm (boxed area). (D) Quantification of the average colony size of colonies shown in Fig. 2. Data are shown as mean±SEM; dots, agar wells; *N*=4. Two-way ANOVA. KO, knockout; *ODF2*, outer dense fiber protein 2; *PLEC*, plectin; PST, plecstatin; WT, wild-type.

**Fig. S5.** Analysis of proteomic signatures of PST treatment, plectin ablation, and ODF2 ablation in SNU-475 cells. (A) Volcano plots show the log2 fold change vs. -log10 of the *p*-value of differentially expressed proteins of the indicated comparisons to WT or *ODF2* KO SNU-475 cells. (B) Venn diagrams show relative proportions of differentially expressed proteins identified by proteomic analysis of WT vs. WT+PST, WT vs. *PLEC* KO, and WT vs. *ODF2* KO SNU-475 cells in cytoskeleton-enriched and cytosolic fractions. KO, knockout; *ODF2*, outer dense fiber protein 2; *PLEC*, plectin; PST, plecstatin; WT, wild-type.

**Fig. S6.** Absence of compensatory effects in *ODF2* KO SNU-475 cells. (A) Principal Component analysis (PCI) in the proteome data sets of WT, WT+PST, *ODF2* KO, and *ODF2* KO+PST SNU- 475 cells in the cytosolic fractions. (B) Volcano plots with depicted ABC efflux pumps in the proteome of *ODF2* KO vs. WT SNU-475 cells. (C) Proteomic expression changes across replicates of untreated and PST-treated WT and *ODF2* KO SNU-475 cells. Log2 fold change values of Label-Free-Quantification (LFQ) protein intensity measurements are shown for depicted proteins across biological replicates of corresponding genotypes. KO, knockout; ODF2, outer dense fiber protein 2; *PLEC*, plectin; PST, plecstatin; WT, wild-type.

**Fig. S7.** Plectin and ODF2 ISR-related signature in HCC patients. The graph shows the association of plectin PLEC (A) and ODF2 (B) mRNA expression with the indicated clinicopathological parameters, molecular classifications, and signature pathways among patients grouped into quartiles of PLEC and ODF2 expression. The analysis is based on data from gene set variation analysis (GSVA) used to generate quantitative enrichment scores for all gene sets from msigdb in pooled and batch-adjusted data. P values represent the result of a chi-square test (for categorical data) or analysis of variance (for numerical data such as gene signature expression levels). HCC, hepatocellular carcinoma; *ODF2*, outer dense fiber protein 2; *PLEC*, plectin.
