# Supplementary figures and images for: Plecstatin inhibits hepatocellular carcinoma tumorigenesis and invasion through cytolinker plectin
Source: Mol Oncol. 2025 Dec 30;20(6):1453–72. doi: 10.1002/1878-0261.70186 (PMC13238617; doi:10.1002/1878-0261.70186)

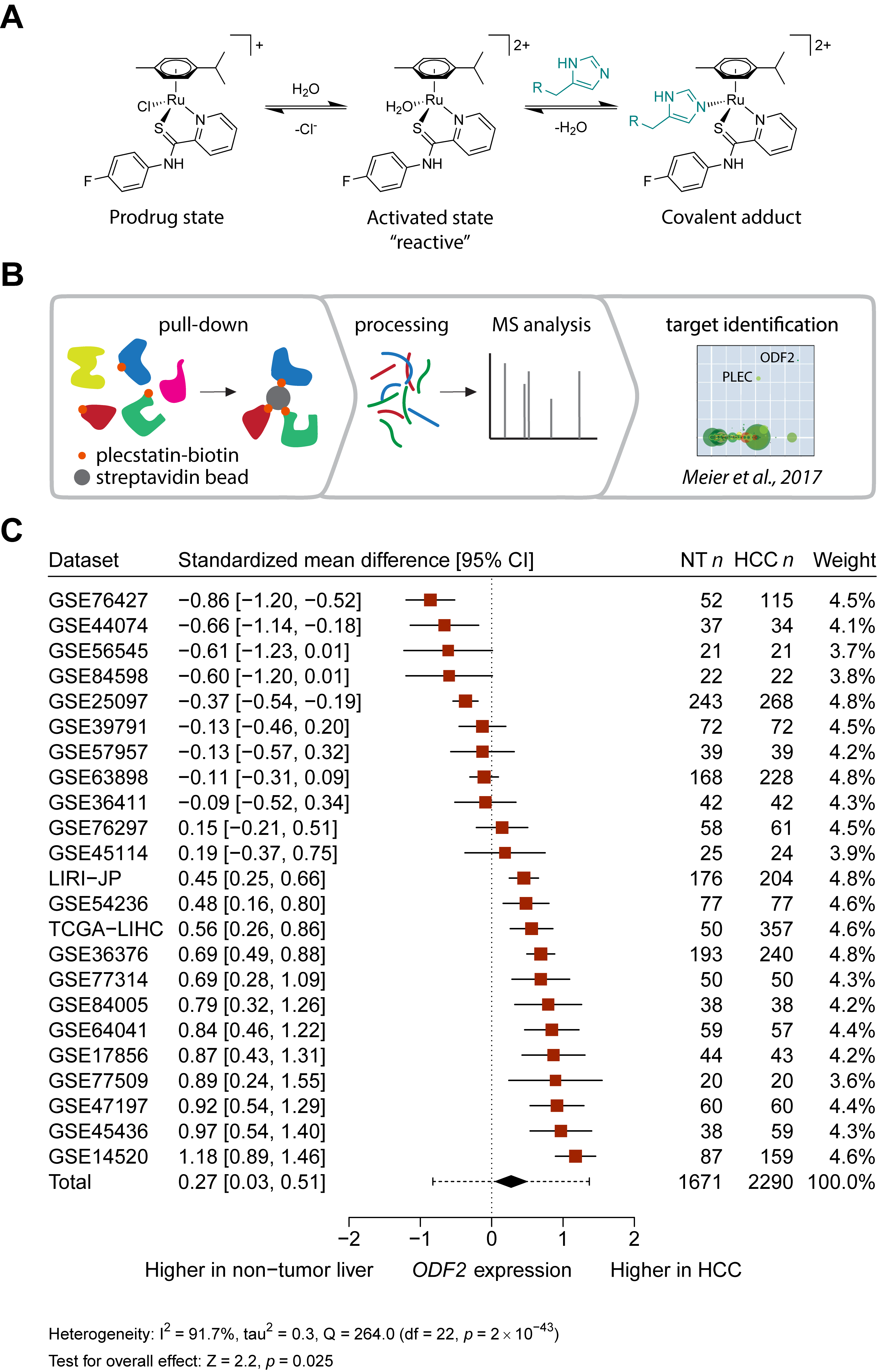

Supplement: Supplementary file 1 — Fig. S1. Plectin and ODF2 targeting by PST and ODF2 expression in hepatocellular carcinoma. Fig. S2. Gene expression patterns of both plectin and ODF2 across the entire HCC tumor microenvironment. Fig. S3. Association of plectin and ODF2 expression with molecular subclasses of HCC and mutations. Fig. S4. Verification of plectin and ODF2 gene depletion in SNU‐475 cell lines and the effect of PST treatment on colony size. Fig. S5. Analysis of proteomic signatures of PST treatment, plectin ablation, and ODF2 ablation in SNU‐475 cells. Fig. S6. Absence of compensatory effects in ODF2 KO SNU‐475 cells. Fig. S7. Plectin and ODF2 ISR‐related signature in HCC patients. Table S1. Primary and secondary antibodies used in this study. Table S2. The classification of 23 HCC cell lines using RNA expression‐based nearest template prediction (NTP) into Dr Boyault's molecular subgroups (G1–G6) of HCC. [file MOL2-20-1453-s001.zip › mol270186-sup-0002-FigureS1.tif]

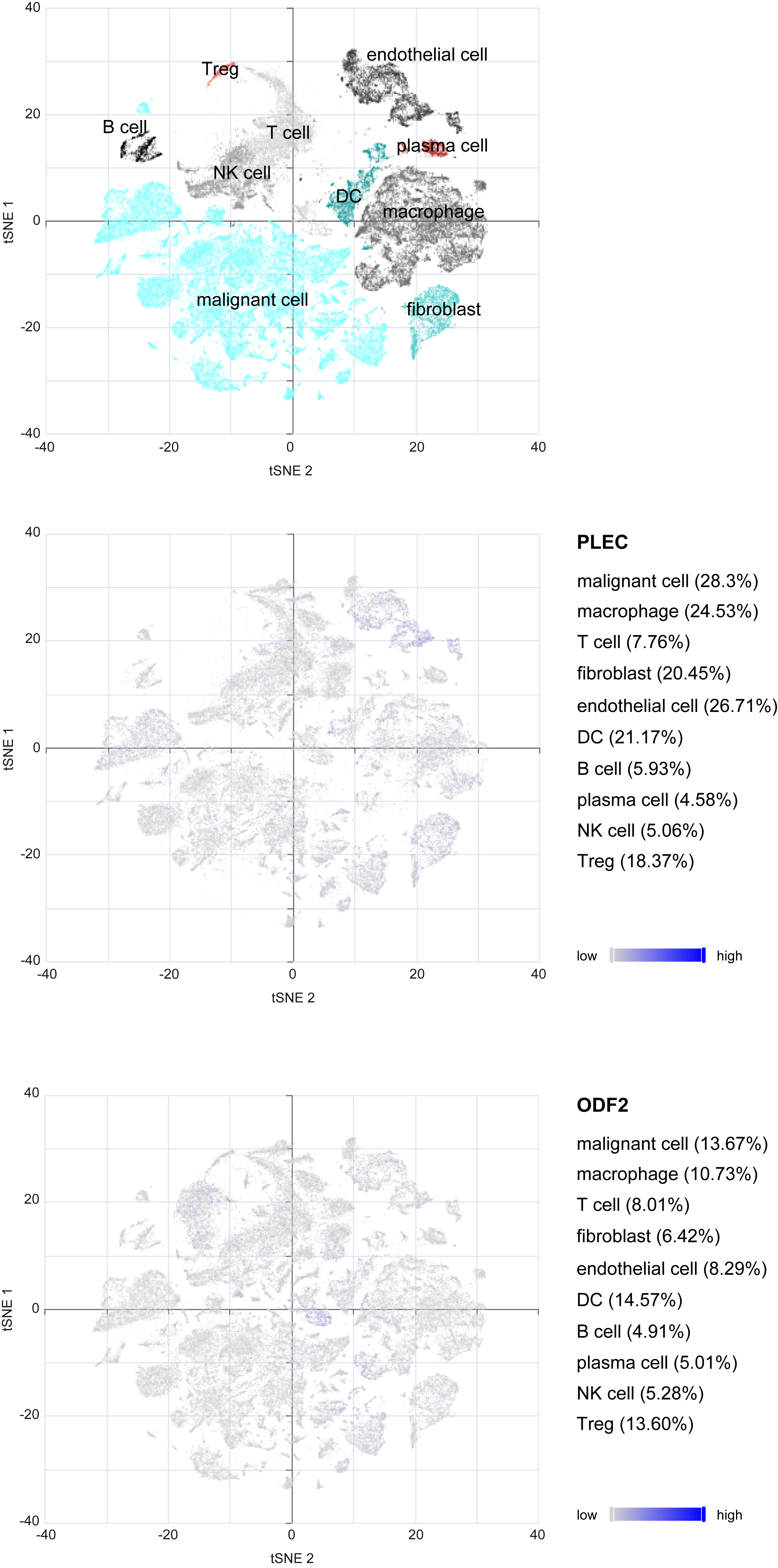

Supplement: Supplementary file 1 — Fig. S1. Plectin and ODF2 targeting by PST and ODF2 expression in hepatocellular carcinoma. Fig. S2. Gene expression patterns of both plectin and ODF2 across the entire HCC tumor microenvironment. Fig. S3. Association of plectin and ODF2 expression with molecular subclasses of HCC and mutations. Fig. S4. Verification of plectin and ODF2 gene depletion in SNU‐475 cell lines and the effect of PST treatment on colony size. Fig. S5. Analysis of proteomic signatures of PST treatment, plectin ablation, and ODF2 ablation in SNU‐475 cells. Fig. S6. Absence of compensatory effects in ODF2 KO SNU‐475 cells. Fig. S7. Plectin and ODF2 ISR‐related signature in HCC patients. Table S1. Primary and secondary antibodies used in this study. Table S2. The classification of 23 HCC cell lines using RNA expression‐based nearest template prediction (NTP) into Dr Boyault's molecular subgroups (G1–G6) of HCC. [file MOL2-20-1453-s001.zip › mol270186-sup-0003-FigureS2.tif]

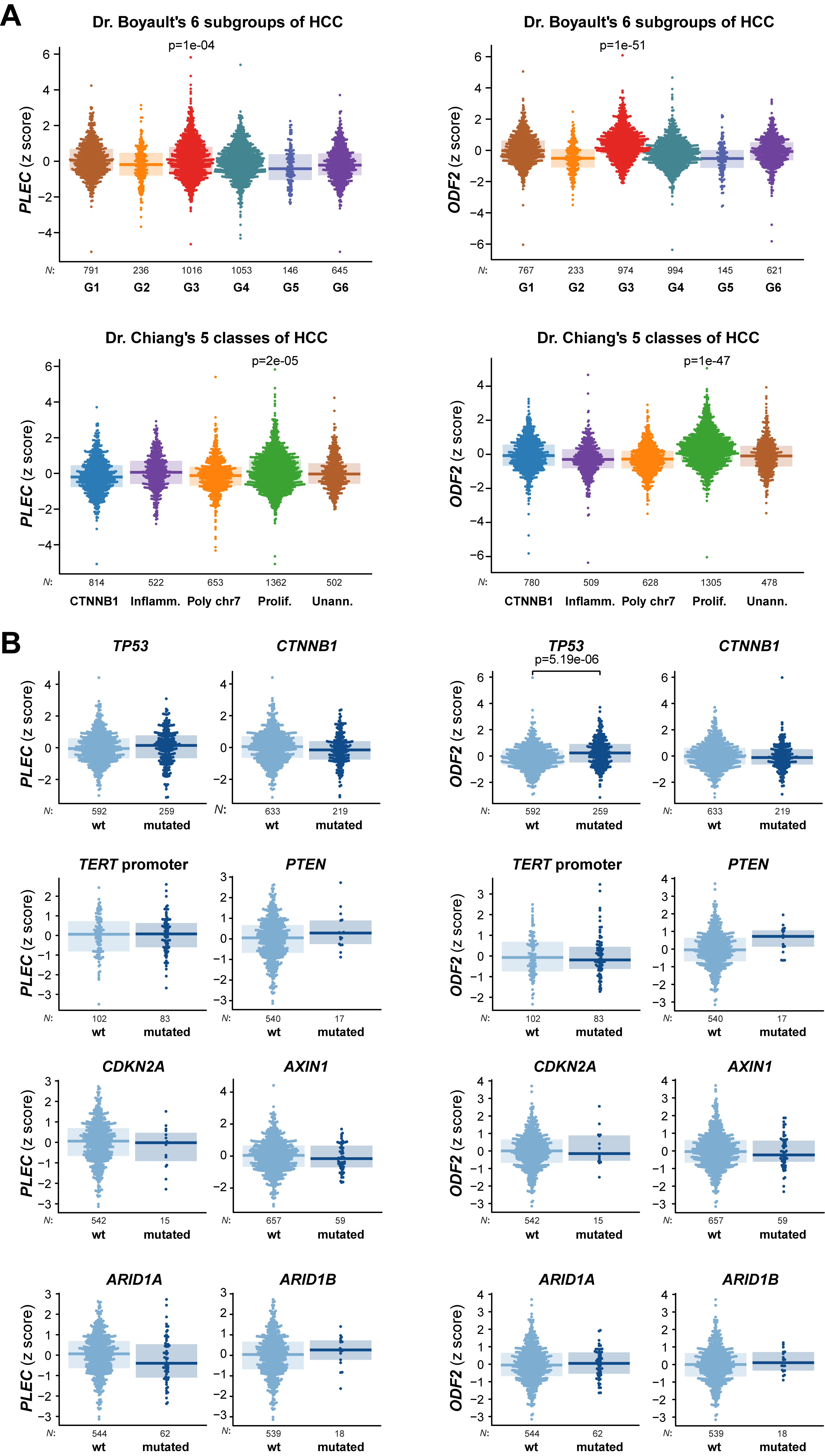

Supplement: Supplementary file 1 — Fig. S1. Plectin and ODF2 targeting by PST and ODF2 expression in hepatocellular carcinoma. Fig. S2. Gene expression patterns of both plectin and ODF2 across the entire HCC tumor microenvironment. Fig. S3. Association of plectin and ODF2 expression with molecular subclasses of HCC and mutations. Fig. S4. Verification of plectin and ODF2 gene depletion in SNU‐475 cell lines and the effect of PST treatment on colony size. Fig. S5. Analysis of proteomic signatures of PST treatment, plectin ablation, and ODF2 ablation in SNU‐475 cells. Fig. S6. Absence of compensatory effects in ODF2 KO SNU‐475 cells. Fig. S7. Plectin and ODF2 ISR‐related signature in HCC patients. Table S1. Primary and secondary antibodies used in this study. Table S2. The classification of 23 HCC cell lines using RNA expression‐based nearest template prediction (NTP) into Dr Boyault's molecular subgroups (G1–G6) of HCC. [file MOL2-20-1453-s001.zip › mol270186-sup-0004-FigureS3.tif]

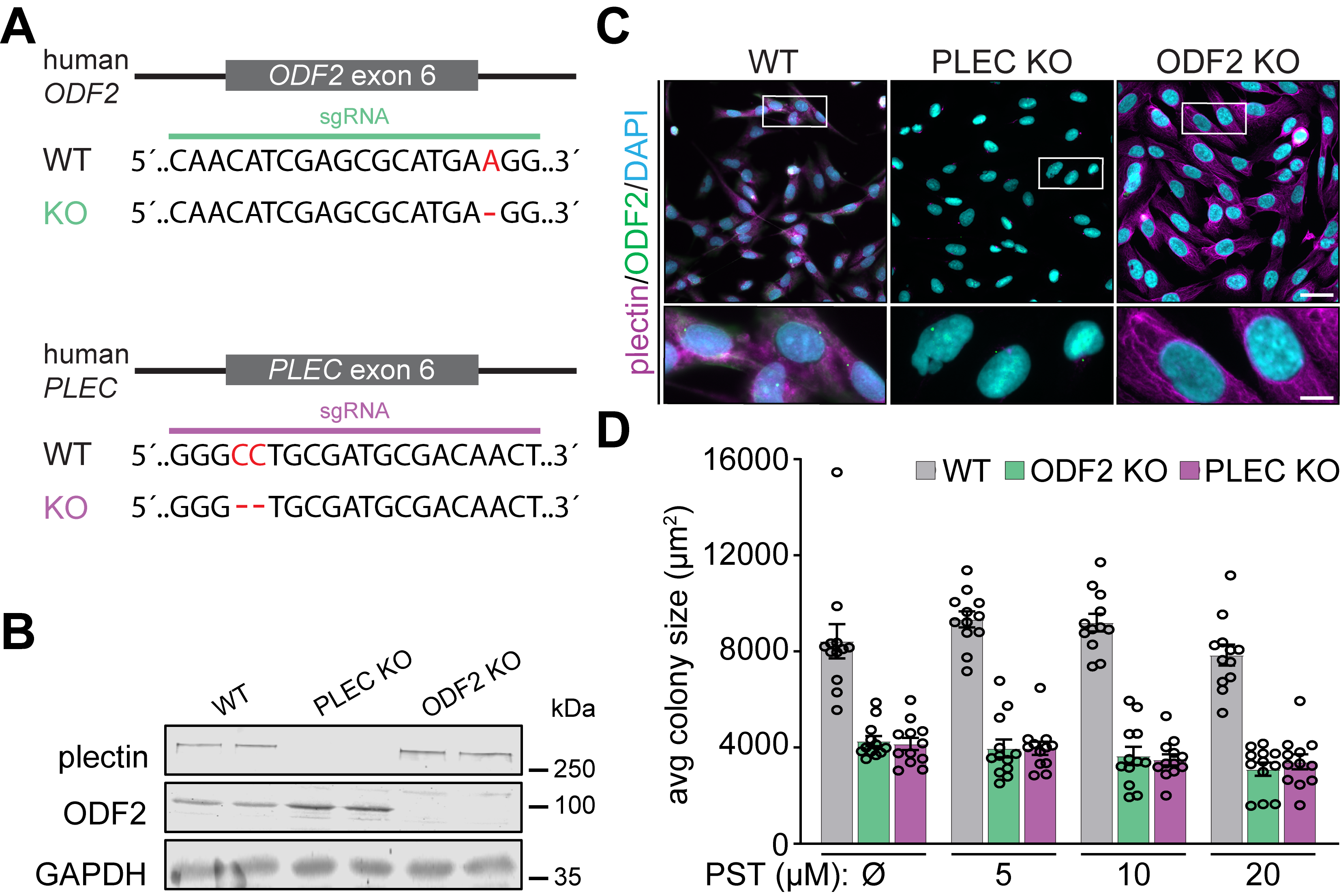

Supplement: Supplementary file 1 — Fig. S1. Plectin and ODF2 targeting by PST and ODF2 expression in hepatocellular carcinoma. Fig. S2. Gene expression patterns of both plectin and ODF2 across the entire HCC tumor microenvironment. Fig. S3. Association of plectin and ODF2 expression with molecular subclasses of HCC and mutations. Fig. S4. Verification of plectin and ODF2 gene depletion in SNU‐475 cell lines and the effect of PST treatment on colony size. Fig. S5. Analysis of proteomic signatures of PST treatment, plectin ablation, and ODF2 ablation in SNU‐475 cells. Fig. S6. Absence of compensatory effects in ODF2 KO SNU‐475 cells. Fig. S7. Plectin and ODF2 ISR‐related signature in HCC patients. Table S1. Primary and secondary antibodies used in this study. Table S2. The classification of 23 HCC cell lines using RNA expression‐based nearest template prediction (NTP) into Dr Boyault's molecular subgroups (G1–G6) of HCC. [file MOL2-20-1453-s001.zip › mol270186-sup-0005-FigureS4.tif]

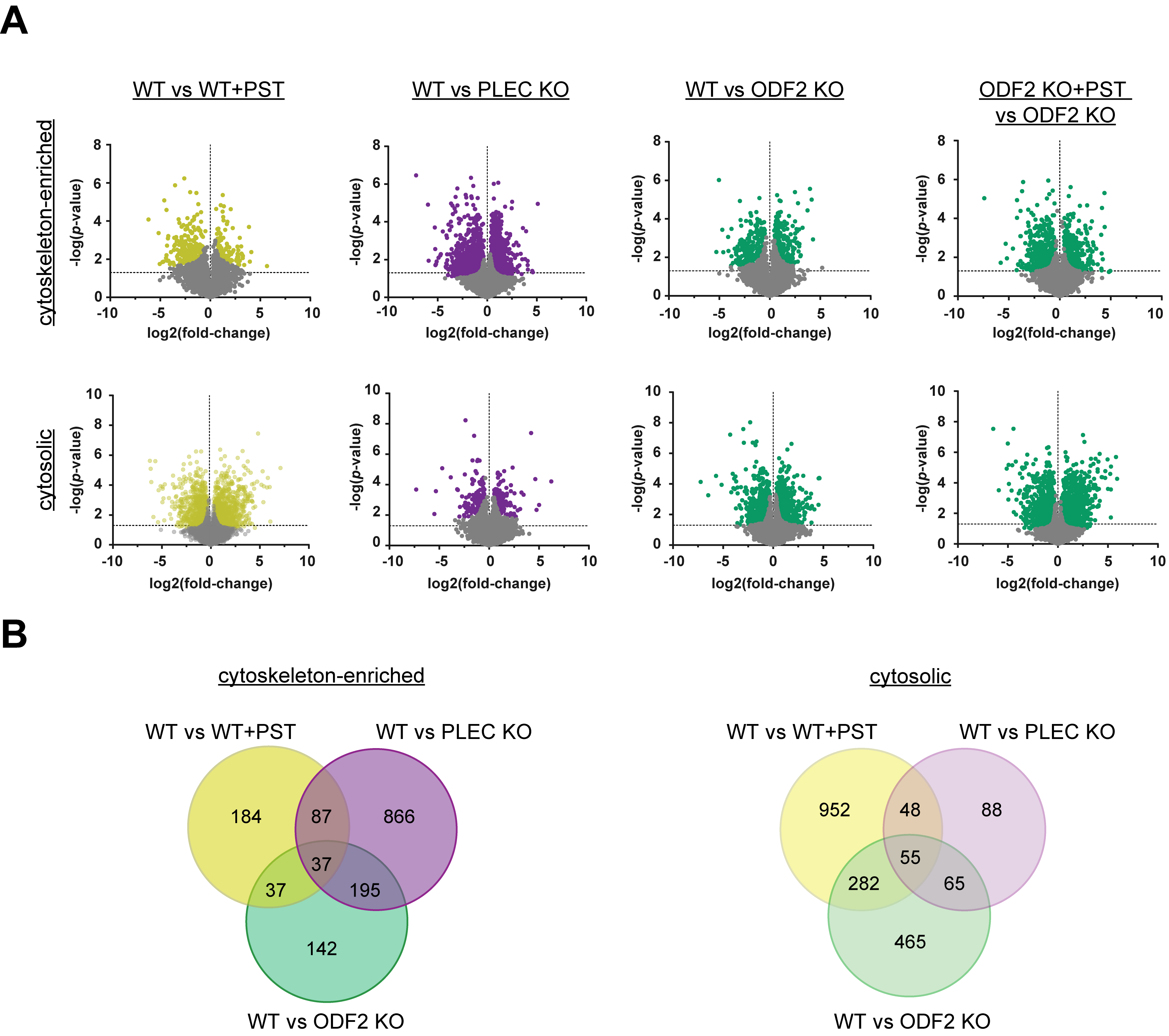

Supplement: Supplementary file 1 — Fig. S1. Plectin and ODF2 targeting by PST and ODF2 expression in hepatocellular carcinoma. Fig. S2. Gene expression patterns of both plectin and ODF2 across the entire HCC tumor microenvironment. Fig. S3. Association of plectin and ODF2 expression with molecular subclasses of HCC and mutations. Fig. S4. Verification of plectin and ODF2 gene depletion in SNU‐475 cell lines and the effect of PST treatment on colony size. Fig. S5. Analysis of proteomic signatures of PST treatment, plectin ablation, and ODF2 ablation in SNU‐475 cells. Fig. S6. Absence of compensatory effects in ODF2 KO SNU‐475 cells. Fig. S7. Plectin and ODF2 ISR‐related signature in HCC patients. Table S1. Primary and secondary antibodies used in this study. Table S2. The classification of 23 HCC cell lines using RNA expression‐based nearest template prediction (NTP) into Dr Boyault's molecular subgroups (G1–G6) of HCC. [file MOL2-20-1453-s001.zip › mol270186-sup-0006-FigureS5.tif]

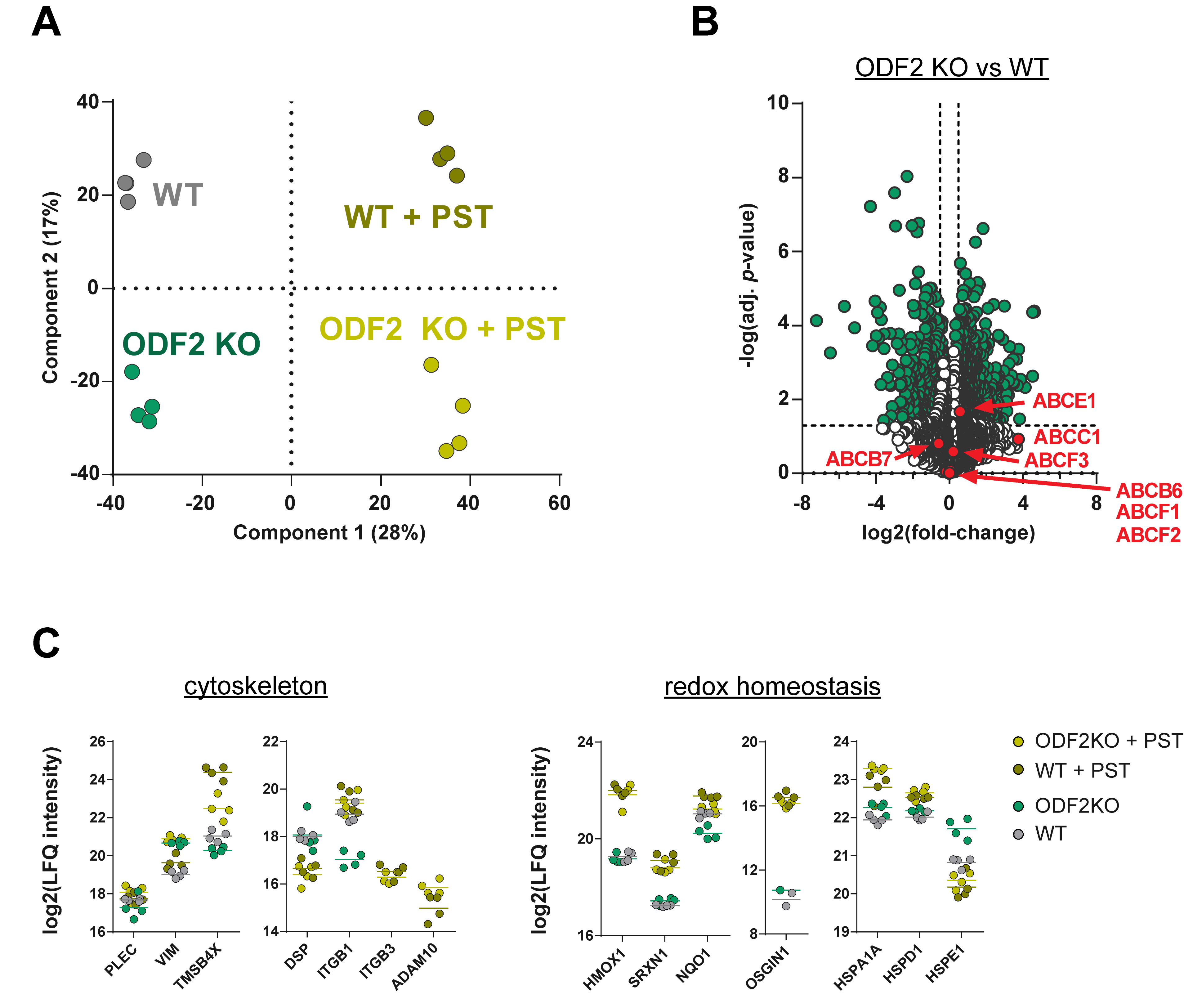

Supplement: Supplementary file 1 — Fig. S1. Plectin and ODF2 targeting by PST and ODF2 expression in hepatocellular carcinoma. Fig. S2. Gene expression patterns of both plectin and ODF2 across the entire HCC tumor microenvironment. Fig. S3. Association of plectin and ODF2 expression with molecular subclasses of HCC and mutations. Fig. S4. Verification of plectin and ODF2 gene depletion in SNU‐475 cell lines and the effect of PST treatment on colony size. Fig. S5. Analysis of proteomic signatures of PST treatment, plectin ablation, and ODF2 ablation in SNU‐475 cells. Fig. S6. Absence of compensatory effects in ODF2 KO SNU‐475 cells. Fig. S7. Plectin and ODF2 ISR‐related signature in HCC patients. Table S1. Primary and secondary antibodies used in this study. Table S2. The classification of 23 HCC cell lines using RNA expression‐based nearest template prediction (NTP) into Dr Boyault's molecular subgroups (G1–G6) of HCC. [file MOL2-20-1453-s001.zip › mol270186-sup-0007-FigureS6.tif]

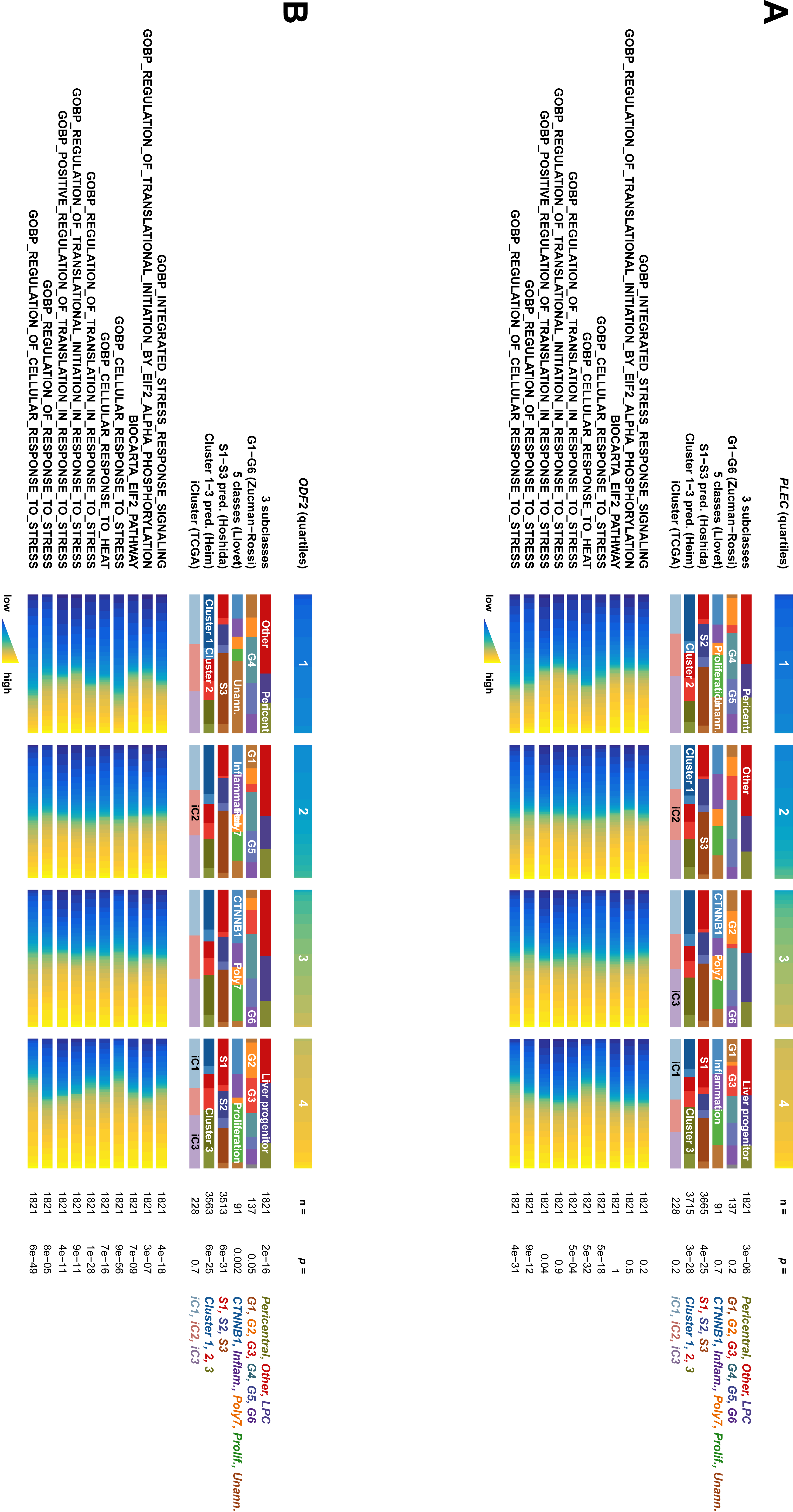

Supplement: Supplementary file 1 — Fig. S1. Plectin and ODF2 targeting by PST and ODF2 expression in hepatocellular carcinoma. Fig. S2. Gene expression patterns of both plectin and ODF2 across the entire HCC tumor microenvironment. Fig. S3. Association of plectin and ODF2 expression with molecular subclasses of HCC and mutations. Fig. S4. Verification of plectin and ODF2 gene depletion in SNU‐475 cell lines and the effect of PST treatment on colony size. Fig. S5. Analysis of proteomic signatures of PST treatment, plectin ablation, and ODF2 ablation in SNU‐475 cells. Fig. S6. Absence of compensatory effects in ODF2 KO SNU‐475 cells. Fig. S7. Plectin and ODF2 ISR‐related signature in HCC patients. Table S1. Primary and secondary antibodies used in this study. Table S2. The classification of 23 HCC cell lines using RNA expression‐based nearest template prediction (NTP) into Dr Boyault's molecular subgroups (G1–G6) of HCC. [file MOL2-20-1453-s001.zip › mol270186-sup-0008-FigureS7.tif]
